# Differential Evolution of CDS and UTR Non-canonical RNA G-quadruplex Structures in Eukaryotic Transcriptomes

**DOI:** 10.1093/gpbjnl/qzaf078

**Published:** 2025-09-14

**Authors:** Eugene Yui-Ching Chow, Jieyu Zhao, Chun Kit Kwok, Ting-Fung Chan

**Affiliations:** School of Life Sciences, and State Key Laboratory of Agrobiotechnology, The Chinese University of Hong Kong, Hong Kong Special Administrative Region 999077, China; Department of Chemistry and State Key Laboratory of Marine Pollution, City University of Hong Kong, Hong Kong Special Administrative Region 999077, China; Department of Chemistry and State Key Laboratory of Marine Pollution, City University of Hong Kong, Hong Kong Special Administrative Region 999077, China; Shenzhen Research Institute of City University of Hong Kong, Shenzhen 518057, China; School of Life Sciences, and State Key Laboratory of Agrobiotechnology, The Chinese University of Hong Kong, Hong Kong Special Administrative Region 999077, China

**Keywords:** RNA G-quadruplex, RNA structure, Evolutionary and computational biology, Transcriptomics, Comparative genomics

## Abstract

RNA G-quadruplexes (rG4s) are non-classical, four-stranded secondary RNA structures that play regulatory roles in various biological processes. Although canonical rG4s have been studied extensively, recent advancements have underscored the importance of non-canonical rG4s. In this study, we experimentally determined rG4 structures from multiple eukaryotic species. Bioinformatic analysis revealed that across 1 billion years of evolution, rG4s have comprised an integral feature of eukaryotic transcriptomes; additionally, non-canonical rG4s consistently were found to dominate the surveyed rG4omes. Over time, the overall size of the rG4ome has expanded progressively, accompanied by a notable compositional shift such that untranslated region (UTR) rG4s became favored over protein coding sequence (CDS) rG4s. Additionally, we observed distinct evolutionary patterns for CDS and UTR rG4s, which involved differential evolutionary origins and canonicality drift patterns. Our findings suggest that new UTR rG4 sequences emerge rapidly during early mammalian evolution, whereas the more gradual increase in CDS rG4s is linked to changes in selective amino acid residue preferences. This plausible theory accounts for both the prevalence of UTR rG4s and the emergence of canonical motifs in mammalian models. Access to all the rG4 structures identified in this study is available through the rG4-seq Database application at https://rg4s.science/.

## Graphical abstract

**Figure:**
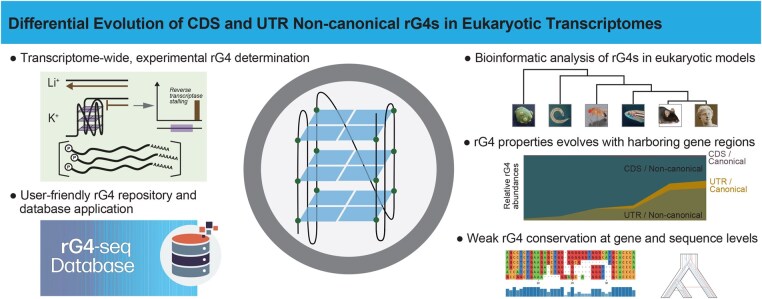

## Introduction

**Figure 1:**
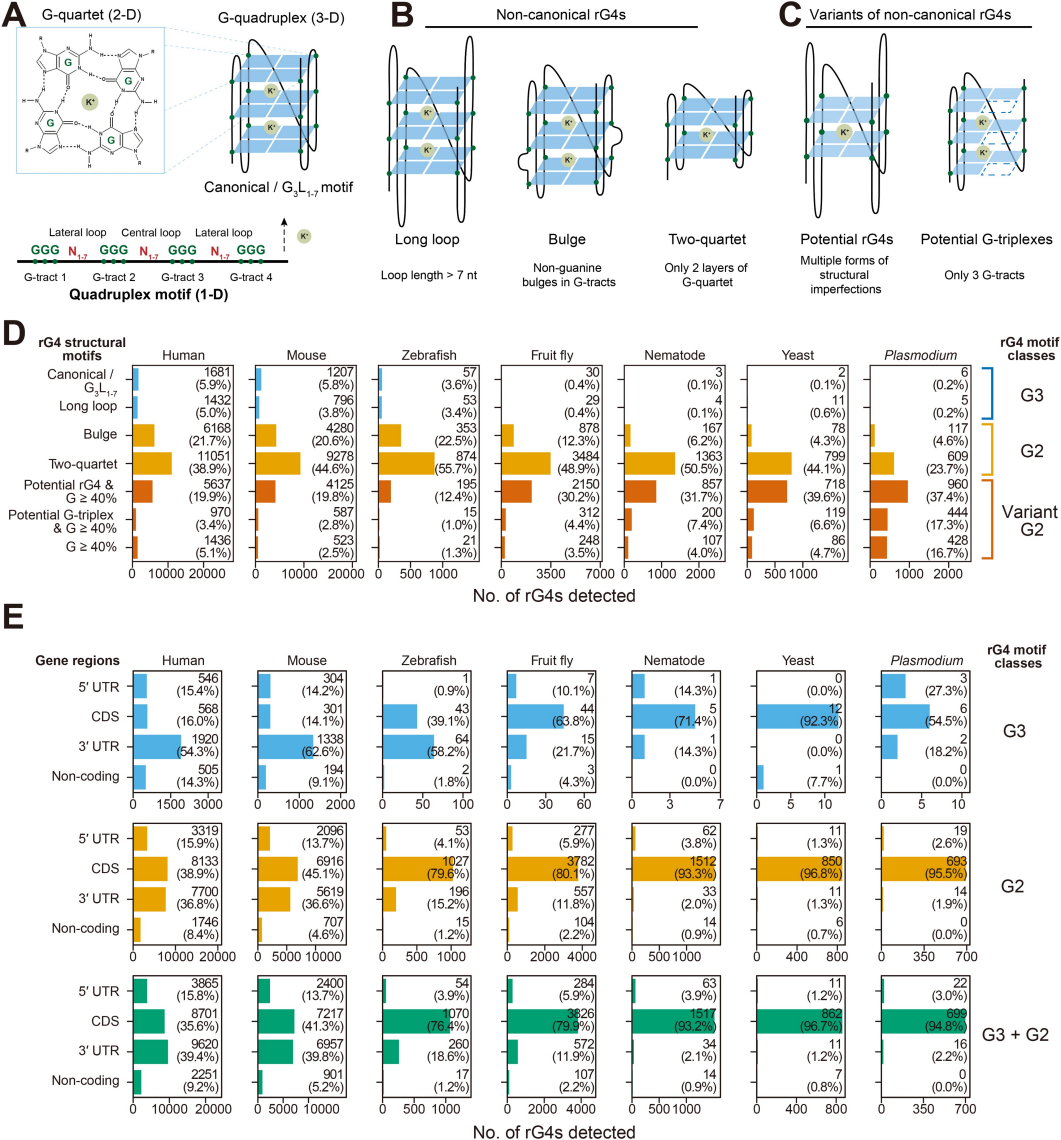
The landscapes of RNA G-quadruplex structures in model species **A**. Structural arrangement of a canonical rG4 in three dimensions. **B**. and **C**. Illustrations of the structural features of non-canonical rG4s (B) and variants of non-canonical rG4s (C). **D**. Distribution of rG4s by structural motif. rG4s were further classified as G3, G2, or variant G2 based on their motif definitions and requirements for GGG G-tracts. Only G3 and G2 rG4s were included in subsequent analyses. **E**. Distribution of G3 and G2 rG4s by gene regions. rG4, RNA G-quadruplex; CDS, coding sequence; UTR, untranslated region; GGG, tri-guanine.

RNA G-quadruplexes (rG4s) are non-classical secondary RNA structures formed by guanine-rich sequences. These sequences fold into stable, monovalent cation-coordinated, four-stranded topologies via Hoogsteen hydrogen bonds [1]. In an rG4 structure, short extensions of guanine residues (*i.e.*, G-tracts) interleaved by arbitrary residues (*i.e.*, loops) interact to form planar G-quartets, which stack in two or more layers [2] (**Figure 1**A). rG4s have been discovered in all kingdoms of life, including animals [3], plants [4], bacteria [5], and viruses [6]. The unique three-dimensional conformation and high thermostability of rG4s enable them to play versatile regulatory roles across a wide range of biological functions, including transcription, post-transcription, and translation. The controlled formation and resolution of these functions may be mediated by rG4s [7–9].

rG4s can be subclassified into canonical or non-canonical according to their underlying sequences [10] (Figure 1B and C). Historically, research has focused on canonical rG4s, mainly because their stringent canonical motif has enabled relatively accurate *in silico* prediction from RNA sequences [11,12] and facilitated subsequent functional studies. Recently, however, non-canonical rG4s (NC-rG4s) that can form under more relaxed sequence motif requirements have been reported [13–17]. Despite the interval between the discovery times of both subtypes of rG4s, NC-rG4s should not be considered to have less biological significance than canonical rG4s; the rG4 motif classifications have been generalized from experimental studies, rather than provided as strict biophysical definitions, and NC-rG4s have been shown to exhibit regulatory effects and interact with RNA binding proteins in a manner similar to canonical rG4s [3,5,18–21].

Recent methodological advancements have enabled transcriptome-wide rG4 mapping experiments that are unaffected by the underlying rG4 motifs [3,4,18,22], and these experiments have addressed the difficulties of discovering NC-rG4s under the orthodox prediction-then-validation paradigm [10]. These studies have provided empirical evidence that NC-rG4s are the dominant rG4s in multiple biological systems [3,5,18–21]. In particular, our previous studies have established the prevalence and significance of NC-rG4s in two distinct eukaryotic model organisms, namely humans [3,18] and *Plasmodium* [19]. Another recent study was performed using a yeast model [22]. Together, the findings of these studies suggest that significant transitions occurred in the rG4 landscape across the transcriptomes of primitive and advanced eukaryotes. Notably, in primitive eukaryotes, canonical rG4s appear to have been depleted, and the overall number of rG4s detected is substantially lower than that among advanced eukaryotes [19,22].

Several recent studies have further explored the evolutionary conservation of DNA G4 (dG4) [23–26] and rG4 motifs [27,28]. Despite only using computationally predicted G4 motifs and primarily focusing on canonical G4s, these studies have successfully identified candidate conserved G4 elements suitable for functional characterization [23,24,27]. In addition, given the similarities in motif sequences between dG4 and rG4 [10], some lessons from these studies can be extrapolated to rG4s, including the observation that G4 motifs are generally poorly conserved [26], except for those with shorter loops [24]; the tendency for G4 motifs to be enriched within gene regions [24], and the lack of apparent connections between the genomic GC ratio and G4 abundance [25]. Nevertheless, although these divergent observations suggest that the rG4 family is dynamic and contains rapidly evolving RNA elements, it remains uncertain whether genuine rG4s exhibit the same evolutionary patterns as predicted rG4s.

In this study, we integrated empirically determined rG4s, namely NC-rG4s, which covered a broad timeframe of eukaryotic evolution, to support a comprehensive computational analysis that elucidated the evolutionary landscape of rG4s. By considering NC-rG4s, we obtained new clues regarding the answers to several pending questions, including the timeline of general changes in the abundance, distribution, and canonicality of rG4s; the origins of rG4s in untranslated regions (UTRs) that appear to have been depleted in primitive eukaryotes; and possible drifting between canonical and non-canonical motifs through codon and nucleotide sequence mutations that have preserved the tendency toward quadruplex structure formation.

## Results

### Distinct landscapes of rG4s between primitive and advanced eukaryotic transcriptomes

In this study, new rG4-seq experiments involving selected eukaryotic models were performed at three different buffer conditions: Li^+^ buffer (rG4-non-stabilizing), K^+^ buffer (rG4-stabilizing), and K^+^ buffer with the addition of the G-quadruplex stabilizing small molecule pyridostatin (PDS) (rG4-stabilizing) (**Table 1**). Bioinformatic analysis using the rG4-seeker pipeline was performed to identify rG4s that would not fold in Li^+^ buffer conditions but would form under K^+^ buffer or K^+^/PDS buffer conditions. The detection results were then combined to establish species-specific rG4 structomes for downstream analysis (see Materials and methods section).

**Table 1:** Biological samples used for rG4-seq in this study

Species	Sample	rG4-seq condition
Human (*Homo sapiens*)	Cell lines (HeLa, K562)	Li^+^, K⁺, and K⁺/PDS
Mouse (*Mus musculus*)	Cell line (Bruce4 mESC)	Li^+^, K⁺, and K⁺/PDS
Zebrafish (*Danio rerio*)	Whole organism	Li^+^, K⁺, and K⁺/PDS
Fruit fly (*Drosophila melanogaster*)	Cell line (S2)	Li^+^, K⁺, and K⁺/PDS
Nematode (*Caenorhabditis elegans*)	Whole organism, mixed stage (N2)	Li^+^, K⁺, and K⁺/PDS
Yeast (*Saccharomyces cerevisiae*)	Cell line (BY4741)	Li^+^, K⁺, and K⁺/PDS
*Plasmodium* (*Plasmodium falciparum*)	Whole organism, mixed stage (3D7)	Li^+^, K⁺, and K⁺/PDS

*Note*: rG4-seq, rG4 sequencing; PDS, pyridostatin.

The rG4 profiling results suggested that the rG4 landscapes varied widely among the surveyed eukaryotic species. The between-species differences included the overall number of rG4s, the compositions of rG4 structural motifs, and the locations of rG4 on genes (Figure 1D and E; Table S1). A general increase in the number of rG4 structures with evolutionary progress was observed, except in zebrafish. Among these, the G2 and variant G2 motifs, which possess two layers of G-quartets, were identified as the dominant types of rG4s across all surveyed species. In contrast, the G3 rG4 motif, which has three layers of G-quartets (canonical/G_3_L_1-7_ and long loop), comprised a minority of rG4s in the rG4omes of humans, mice, and zebrafish while being nearly absent among the primitive eukaryotes (Figure 1D).

In addition, rG4s were found to be prevalent in the UTR region in humans and mice but not in other species (Figure 1E), suggesting that the expansion of UTR rG4s may have been a recent evolutionary event that occurred in a subset of eukaryotes. The results showed that rG4s had been an integral feature of eukaryotic transcriptomes across 1 billion years of evolution. Nevertheless, the prevalence of UTR rG4s, the emergence of G3 motifs, and the expansion of the rG4ome size were found to be unique in advanced eukaryotes. In primitive eukaryotes, the rG4omes appeared to be dominated by non-canonical G2 motifs within coding regions.

Given the potential for rG4s to influence nearby microRNA interactions and splicing [10,29], we conducted an analysis intersecting human, mouse, and fruit fly 3′ UTR rG4s with predicted microRNA binding sites from the TargetScan database [30]. Additionally, we identified the subset of rG4s overlapping with splice junctions. Statistics (Tables S2 and S3) and lists of the associated rG4s (Files S1 and S2) are provided as additional information.

### Few coding sequence (CDS) rG4s are associated with DNA repetitive elements or peptide low-complexity regions

**Figure 2:**
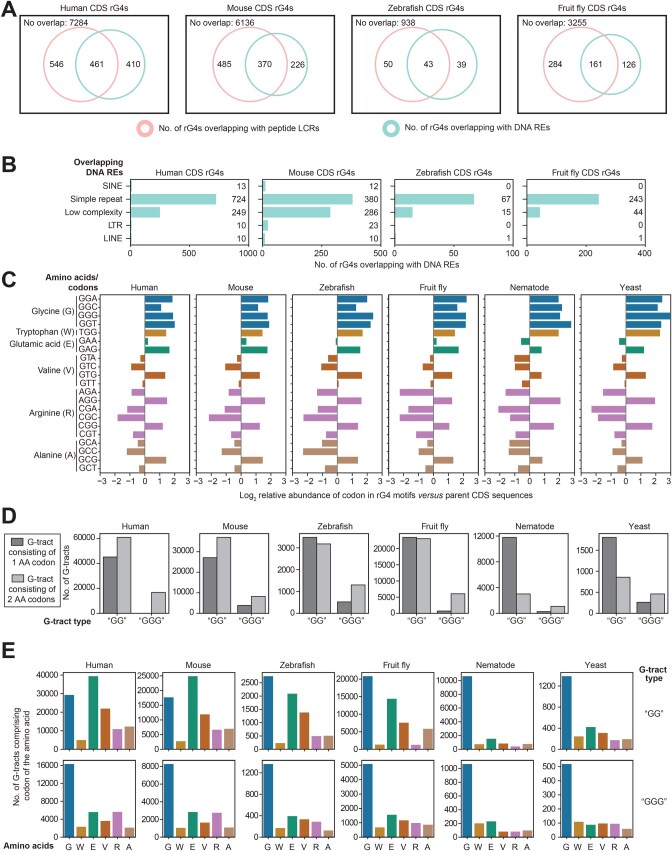
The relationships of rG4s with DNA REs and peptide LCRs **A**. Overlapping of CDS rG4s with peptide LCRs and DNA REs. The vast majority of rG4s do not overlap with either element. **B**. Breakdown of DNA REs overlapping with CDS rG4s. **C**. Enrichment of selective amino acids/codons within CDS rG4 motifs. The CDS sequences of CDS rG4-harboring genes served as the baseline for codon frequencies. Only amino acids with one or more codons having ≥ 2× enrichments are shown in the panel. **D**. Breakdown of CDS rG4 G-tracts by the lengths of the codons they composed. A (GG) G-tract can be composed of one codon or two consecutive codons that end/start with a G. Similarly, a (GGG) G-tract can be composed of either the guanine codon of GGG or a selective combination of two consecutive codons. **E**. Underlying amino acid compositions of the CDS rG4 G-tracts. Only amino acid residues shown to be enriched in (C) are shown here. LCR, low-complexity region; RE, repetitive element; GG, di-guanines.

Our previous analysis of the *Plasmodium* rG4ome suggested that many coding sequence rG4s are associated with DNA repetitive elements (REs) or peptide low-complexity regions (LCRs) [19]. These phenomena were related to local spikes in GC content and may have facilitated rG4 formation. We repeated this analysis using the rG4omes of the six species surveyed (human, mouse, zebrafish, fruit fly, nematode, and yeast) and discovered that only a few CDS rG4s overlapped with repetitive sequences (**Figure 2**A). Nevertheless, as the underlying DNA REs preferred GC-rich, simple DNA repeats with periodicities of three, six, or nine nucleotides, the findings motivated further explorations into the underlying amino acid (AA)/codon compositions of rG4s (Figure 2B; **Table 2**).

**Table 2:** Most common (top 10) DNA repetitive element motifs overlapping with rG4s in human, mouse, zebrafish, and fruit fly

Species	DNA repetitive element type	Repetitive motif	No. of overlapping rG4s
Human	Low complexity	GA-rich	200
Human	Simple repeat	(TCC)_*n*_	85
Human	Simple repeat	(CCT)_*n*_	65
Human	Simple repeat	(CTC)_*n*_	61
Human	Simple repeat	(GGC)_*n*_	40
Human	Low complexity	G-rich	37
Human	Simple repeat	(CCG)_*n*_	36
Human	Simple repeat	(GGA)_*n*_	26
Human	Simple repeat	(GAG)_*n*_	24
Human	Simple repeat	(AGG)_*n*_	16
Mouse	Simple repeat	(GGA)_*n*_	164
Mouse	Low complexity	CT-rich	104
Mouse	Low complexity	GA-rich	101
Mouse	Simple repeat	(TCC)_*n*_	85
Mouse	Simple repeat	(CCG)_*n*_	43
Mouse	Low complexity	GC-rich	41
Mouse	Simple repeat	(CGG)_*n*_	32
Mouse	Low complexity	C-rich	27
Mouse	Simple repeat	(TGG)_*n*_	16
Mouse	Simple repeat	(CCA)_*n*_	12
Zebrafish	Low complexity	GA-rich	13
Zebrafish	Simple repeat	(GGT)_*n*_	7
Zebrafish	Simple repeat	(CCT)_*n*_	6
Zebrafish	Simple repeat	(TGG)_*n*_	5
Zebrafish	Simple repeat	(CCA)_*n*_	5
Zebrafish	Simple repeat	(AGGTGG)_*n*_	5
Zebrafish	Simple repeat	(TCC)_*n*_	4
Zebrafish	Simple repeat	(GGAGGT)_*n*_	3
Zebrafish	Simple repeat	(CTC)_*n*_	3
Zebrafish	Simple repeat	(AGGCGGAGC)_*n*_	2
Zebrafish	Simple repeat	(CCTCTT)_*n*_	2
Zebrafish	Simple repeat	(GGTGGA)_*n*_	2
Zebrafish	Simple repeat	(GGC)_*n*_	2
Zebrafish	Simple repeat	(GATGAG)_*n*_	2
Fruit fly	Low complexity	GA-rich	37
Fruit fly	Simple repeat	(TCC)_*n*_	27
Fruit fly	Simple repeat	(CCA)_*n*_	24
Fruit fly	Simple repeat	(CTC)_*n*_	13
Fruit fly	Simple repeat	(CCT)_*n*_	11
Fruit fly	Simple repeat	(GGT)_*n*_	11
Fruit fly	Simple repeat	(GCG)_*n*_	8
Fruit fly	Simple repeat	(GGC)_*n*_	8
Fruit fly	Simple repeat	(CCG)_*n*_	6
Fruit fly	Simple repeat	(CGAGGA)_*n*_	6
Fruit fly	Simple repeat	(CCACGA)_*n*_	6

### Distinct patterns of shifts in AA residue/codon compositions underlie the evolution of CDS rG4s

Calculating the relative abundances of codons in rG4 sequences versus the CDS sequences of their parent genes revealed that the codons of six types of AA residues were enriched by at least two times (Figure 2C). Interestingly, the enriched codons all contained at least two guanines, whereas codons with only one guanine were not enriched within the rG4 sequences (Figure 2C). This observation suggests that codon enrichment could be directly related to G-tracts, the formation of which requires consecutive guanines.

To explore this phenomenon further, the compositions of the most prevalent types of G-tracts, namely di-guanines (GG) and tri-guanines (GGG) comprising either one or two AA codons, were surveyed. Across all surveyed species, most tri-guanines were found to comprise two codons (Figure 2D). In contrast, there appeared to be a gradual shift in di-guanine composition from one to two codons as evolution progressed (Figure 2D). This change could be important, as it may have increased the possibility of G-tracts occurring within CDS regions due to combinations of AA codons.

Lastly, the usage of the six enriched AAs within the G-tracts of the detected CDS rG4s was examined. Among the tri-guanines, glycine was the dominant AA across all species. Among the di-guanines, although glycine was dominant in yeast and nematodes, the usage of other AA residues, especially glutamic acid and valine, gradually increased in advanced eukaryotes (Figure 2E).

Interestingly, a similar phenomenon was observed within the peptide LCRs overlapping the CDS rG4s. In primitive eukaryotes, glycine was the major component of LCRs, but in higher eukaryotes, glutamic acid and aspartic acid-rich regions became new, frequent sources of rG4-associated LCRs (**Table 3**). Similarly, the DNA repeats associated with poly-glutamic acid, namely the GA-rich and (CCT)*n* repeats, also became more frequently associated with rG4s in advanced eukaryotes.

**Table 3:** Most common (top 10) LCR types (by AA types involved) overlapping with rG4s in human, mouse, zebrafish, fruit fly, nematode, and yeast

Species	AA types involved in LCR	No. of overlapping rG4s
Human	D, E	114
Human	E	103
Human	G, R	92
Human	G	60
Human	G, S	57
Human	E, G	34
Human	A, G	32
Human	E, R	17
Human	G, P	16
Human	D, E, R	16
Mouse	E	129
Mouse	D, E	94
Mouse	G, R	92
Mouse	G, S	47
Mouse	E, G	40
Mouse	G	36
Mouse	G, P	20
Mouse	E, L	19
Mouse	F, G, R	18
Mouse	A, G	18
Zebrafish	G	61
Zebrafish	G, S	59
Zebrafish	A, G	39
Zebrafish	G, R	38
Zebrafish	G, N	33
Zebrafish	D, E	18
Zebrafish	G, V	14
Zebrafish	G, K	12
Zebrafish	E, R	10
Zebrafish	R, S	8
Fruit fly	G, R	14
Fruit fly	G, S	11
Fruit fly	G	10
Fruit fly	D, E	5
Fruit fly	G, K	5
Fruit fly	G, M	4
Fruit fly	F, G, N	4
Fruit fly	E, V	3
Fruit fly	E, G	3
Fruit fly	E	2
Fruit fly	G, Y	2
Fruit fly	D, G, R, Y	2
Fruit fly	D, K	2
Fruit fly	G, P	2
Fruit fly	D, G, R, S, Y	2
Fruit fly	E, G, V	2
Fruit fly	D, R	2
Nematode	G	27
Nematode	G, R	24
Nematode	G, P	17
Nematode	G, S	15
Nematode	G, Y	15
Nematode	A, G, P	8
Nematode	A, G	7
Nematode	C, G	7
Nematode	F, G	6
Nematode	G, M, P	4
Nematode	G, N, Y	4
Nematode	G, Q	4
Nematode	G, K	4
Yeast	G, R	6
Yeast	G, K	5
Yeast	A, G	4
Yeast	N, S	2
Yeast	A, G, R, S	1
Yeast	L, S	1
Yeast	G, S	1
Yeast	E	1
Yeast	F, G, R	1
Yeast	G, I	1
Yeast	G, N, R	1
Yeast	G, P	1
Yeast	G, R, Y	1
Yeast	A, G, P	1

*Note*: LCR, low-complexity region; AA, amino acid.

Moreover, the findings suggest that, in addition to the well-characterized RGG motifs, glutamic acid-rich regions or poly-glutamic acid sequences could be another source of CDS rG4s that have emerged recently in eukaryotic evolution.

### Older protein-coding gene families experiencing whole-genome duplication tend to harbor more rG4s

**Figure 3:**
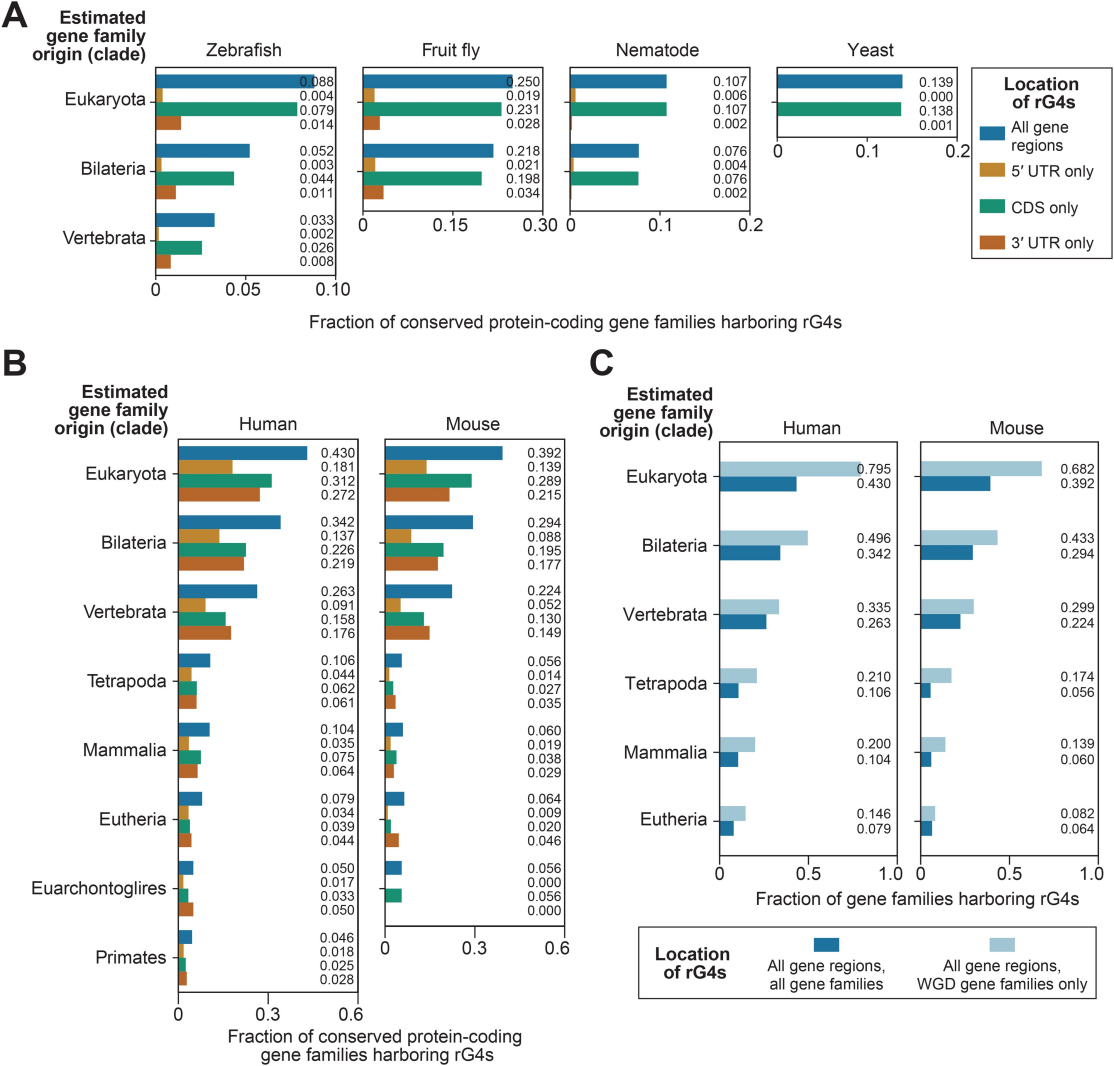
The relationships between the times of origin of conserved protein-coding gene families and their rG4 harboring status **A**. and **B**. Fractions of conserved protein-coding gene families harboring rG4s in the six model species. Different colored bars correspond to the rG4 harboring status when considering all rG4s or only rG4s within the 5′ UTR/CDS/3′ UTR regions. **C**. Comparison of the rG4 harboring status across all protein-coding gene families, and the subset of gene families duplicated during the 2R-WGD at the base of vertebrates. 2R-WGD, two rounds of whole-genome duplication.

To characterize the evolutionary landscape of rG4s at a gene-family level, conserved protein-coding gene families were identified using gene family data and species phylogenetic information downloaded from Ensembl and TimeTree, respectively. The information enabled the estimation of the evolutionary ages of gene families from their representative evolutionary clades. The results showed that older gene families belonging to the Eukaryota, Bilateria, and Vertebrata clades were more likely to harbor rG4s than the younger ones (**Figure 3**A and B). Moreover, human and mouse gene families that contain Ohnolog genes (genes retained from whole-genome duplication in vertebrates) were found to have an even greater tendency to harbor rG4s (Figure 3C). As the observed correlation between gene age and rG4 possession applied to all CDS and UTR regions, and the UTRs of primitive eukaryotes contained very few rG4s, the results suggest that many rG4s in advanced eukaryotes might have been inserted into the transcriptome after gene birth. Compared with younger genes, older genes appeared to have a greater chance of acquiring rG4s in this evolutionary process.

### CDS rG4s are not strongly conserved across long-term eukaryotic evolution

**Figure 4:**
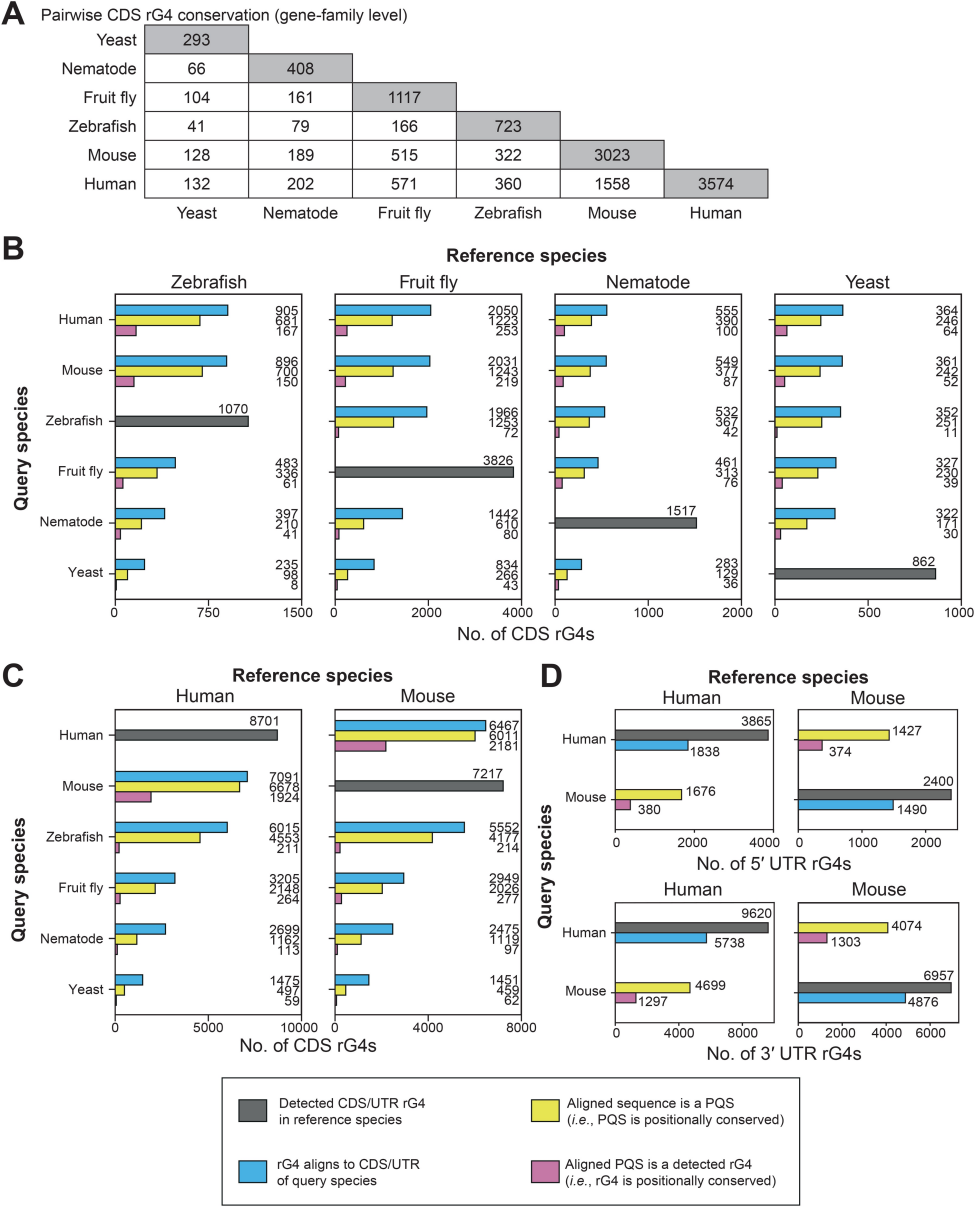
The conservation of rG4 motifs at the gene-family and position levels **A**. Breakdown of the pairwise conservation of CDS rG4 harboring gene families across the surveyed model species. **B**. and **C**. Numbers of pairwise, positionally conserved CDS rG4s across the surveyed model species, with zebrafish, fruit fly, nematode, and yeast in (B), and human and mouse in (C). **D**. Number of pairwise, positionally conserved UTR rG4s between human and mouse; PQS, putative quadruplex sequence.

To further investigate the long-term conservation of CDS rG4s, the rG4s detected on members of conserved gene families were polled and mapped onto the transcript loci of rG4 motifs using multiple alignments of transcript sequences. At a gene-family level, no clear pattern of rG4 conservation could be identified, as the number of pairwise conserved rG4s between two primitive eukaryotes appeared to be generally lower than that between primitive and advanced eukaryotes (**Figure 4**A). Possibly, many older CDS rG4s were lost during evolution. A recent evolutionary event could have reintroduced new rG4s into genes that previously had harbored rG4s, albeit not necessarily at the same loci. A deeper examination at the position level revealed that some of the CDS rG4s in primitive eukaryotes resided in conserved CDS regions, which were highly enriched for harboring putative quadruplex sequences (PQSs). However, the overall positional conservation at the rG4 structural level was minimal (Figure 4B and C).

In contrast, the number of rG4s found to align with the CDSs and PQSs of more recent species appeared to be relatively stable (Figure 4B and C). This finding could suggest an overlap between more conserved protein subregions and peptide subsequences, such that their codons tend to induce PQSs. However, the folding status of the PQSs might not be strongly conserved, and only a few conserved PQSs would meet the requirement to become conserved rG4s. In contrast, the number of rG4s that aligned with the CDSs and PQSs of older species showed a decreasing trend over time (Figure 4B and C). This observation might be attributable to the finding that advanced eukaryotes use different compositions of amino acid residues to form G-tracts and G4s, as shown in Figure 2E, whereas these newer protein regions might not be present in older species.

### Limited positional conservation of rG4s between humans and mice

A comparison of human and mouse rG4s enabled examination of the positional conservation status of more recent rG4s in both the CDS and UTR regions. Despite the generally high level of conservation of rG4-harboring codon sequences and PQSs in the CDS region, only a minority of rG4 structures were positionally conserved (Figure 4C). In comparison, fewer rG4-harboring UTR regions were found to align between humans and mice, given the higher cross-species variations in UTR sequences. Within these UTR regions, the observations were similar to those in CDS regions: most regions harbored PQSs, but only a minority of rG4 structures among the PQSs were positionally conserved (Figure 4D). The findings suggest that human and mouse rG4s are likely to reside in conserved, PQS-harboring transcriptomic regions that are susceptible to rG4 structure formation. However, the folding statuses of these rG4 structures might not be strongly conserved. The findings motivate us to further explore the aspects of rG4 sequence evolution.

### Defining rG4 canonicality and a classification scheme

**Figure 5:**
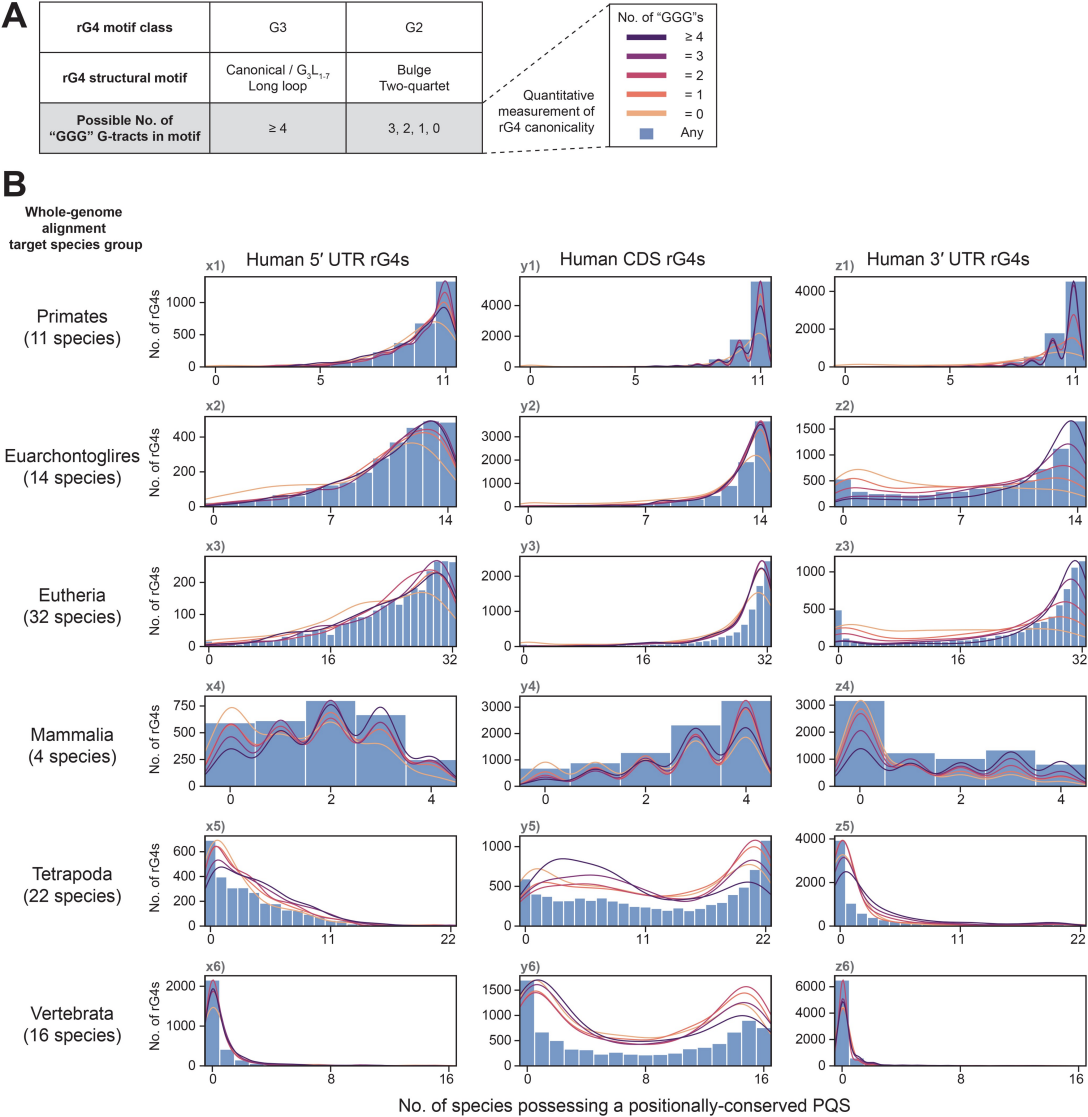
The conservation of the underlying quadruplex sequences of human rG4s across vertebrate species **A**. Scheme to quantify the canonicality (*i.e.*, similarity to a canonical rG4 structural motif) of rG4s by counting the number of (GGG) G-tracts in the quadruplex sequence. The canonical and non-canonical long loop motifs consist of four or more tri-guanine G-tracts. The non-canonical bulges and two-quartet motifs consist of a mixture of (GG) and (GGG) G-tracts, necessitating a finer classification scheme. **B**. Determination of conservation status of the QSs underlying human rG4s using whole-genome multiple alignments. For a given species, if the genomic region aligned with the human rG4 contains a PQS, the species possesses a positionally conserved PQS; thus, the QS underlying the human rG4 is considered conserved. Vertebrate species were further grouped into representative evolutionary clades (in reverse-chronological order) to illustrate the correlations between evolutionary times and QS/PQS conservation status. The member species of each clade and the estimated evolutionary divergence time are shown in Figure S2. rG4s were further grouped by their harboring gene regions (5′ UTR/CDS/3′ UTR) to highlight differences in their conservation statuses. The bar plot shows the overall distribution of the conservation statuses of all rG4s, regardless of their canonicality. The overlaid kernel distribution estimation plot shows the distributions of individual rG4s of different canonicalities. QS, quadruplex sequence.

In many previous rG4 studies, the classification of rG4 structures has depended largely on generalized definitions of structural motifs, which describe rG4s according to the lengths of the four G-tracts and the three connecting loops. Notably, the possession of four tri-guanine (GGG) G-tracts is the hallmark feature of the canonical rG4 motif, defining a subclass of rG4s that can form the most thermodynamically stable three-layered quadruplexes. Meanwhile, the two-quartet rG4 motif was thought to be degenerate and to have the most relaxed criteria, requiring only four (GG) G-tracts to form. Interestingly, many detected rG4s were found to contain a mixture of (GGG) and (GG) G-tracts and thus had an intermediate status; despite being non-canonical, they were more canonical than the two-quartet motif. To better indicate the similarity of rG4s to the canonical motif, *i.e.*, rG4 canonicality, a quantitative scheme was proposed to classify rG4s based on the number of (GGG) G-tracts in the motif (**Figure 5**A), which could range from 0 to 4. For rare cases where (GGG) G-tracts exceed 4, they were classified together with 4. Re-classifying the detected rG4s according to this new canonicality metric revealed some exciting findings (**Table 4**). Among CDS rG4s, while an overall increasing trend in canonicality could be observed across evolution, low-canonicality rG4s remained more dominant.

**Table 4:** Statistics of rG4 detections by gene region and canonicality

	rG4 canonicality / No. of “GGG” s	Human	Mouse	Zebrafish	Fruit fly	Nematode	Yeast
5′ UTR rG4s	4	586	321	1	7	1	0
	3	787	448	11	14	1	0
	2	835	498	16	29	7	2
	1	1018	689	14	97	14	4
	0	639	444	12	137	40	5
CDS rG4s	4	599	315	43	47	5	12
	3	1088	591	131	190	32	66
	2	1654	1197	270	421	110	191
	1	2638	2459	293	1217	396	302
	0	2722	2655	333	1951	974	291
3′ UTR rG4s	4	2075	1432	66	15	1	0
	3	2241	1474	77	43	2	0
	2	2062	1462	52	77	3	4
	1	1939	1418	41	189	9	4
	0	1303	1171	24	248	19	3
Non-coding rG4s	4	537	205	2	3	0	1
	3	503	200	5	7	1	1
	2	502	176	5	11	2	0
	1	399	183	3	39	6	3
	0	310	137	2	47	5	2

*Note:* GGG, tri-guanine.

Moreover, the UTR rG4s in primitive eukaryotes also showed a similar pattern that favored low-canonicality rG4s. In contrast, the UTR rG4s in humans, mice, and zebrafish showed a distinctive pattern that generally favored rG4s with intermediate canonicality (Table 4). The findings highlight that the distinct evolutionary patterns of UTR and CDS rG4s are also reflected in canonicality, with the drift in rG4 canonicality appearing to be a feature of eukaryotic evolution. Crucially, the findings suggest that the influence of rG4 canonicality should be considered when investigating the sequence evolution of rG4s.

### Capturing the conservation landscape of the underlying quadruplex sequences of human and mouse rG4s across vertebrate clades

To further explore the origins and evolutionary dynamics of rG4s at the sequence level, whole-genome multiple alignments of vertebrate species were used to retrieve sequences orthologous to the detected rG4s. PQSs were identified from the orthologous sequences, and the sequence identities between the PQSs and the human/mouse sequence were calculated.

Although the presence of orthologous rG4 structures in other species could be assumed from genomic alignment information alone, the absence of PQSs can be considered to indicate the absence of rG4 structures at specific loci, given that an underlying PQS is a requirement for an rG4 structure.

All species included in the UCSC Multiz 100-way and 60-way multiple alignment data [31] were also segregated into nested, representative taxonomic categories (Primates/Glires, Euarchontoglires, Eutheria, Mammalia, Tetrapoda, and Vertebrata) according to their estimated times of divergence from humans or mice during vertebrate evolution. The time of divergence can provide a rough timescale for any identified evolutionary events of a species (Figures S1 and S2).

### A recent evolutionary event has driven the incorporation of new quadruplex sequences into mammalian genomes

Multiple alignments first revealed that most rG4s, regardless of their locations in transcripts, could be aligned with PQSs from most species in the Primates, Euarchontoglires, and Eutheria categories (Figure 5B, Figure S3: subplots x1–3, y1–3, z1–3). However, for UTR rG4s, the PQS conservation landscape changed drastically in the Mammalia, Tetrapoda, and Vertebrata categories (Figure 5B, Figure S3: subplots x4–6, z4–6): the majority of rG4s could not be aligned with a PQS in most species. The absence of alignable PQSs suggested that the UTR rG4 structures and sequences were unlikely to have been conserved among vertebrates that had diverged earlier. Meanwhile, the changes in the PQS conservation landscape of CDS rG4s appeared to be more gradual, shifting from a right-skewed distribution (Figure 5B, Figure S3: subplots y1–4) to a bimodal distribution (Figure 5B, Figure S3: subplots y5–6). The distributions suggested a mixture of CDS rG4s with more conserved underlying sequences (right-skewed observations) and fewer unconserved CDS rG4s (left-skewed observations) over the timeline of vertebrate evolution. The alignments of mouse and human rG4s revealed very similar PQS evolutionary landscapes, suggesting that the species experienced similar modes of rG4 evolution. Although greater variation in PQS alignments was observed in the Glires than in Primates (Figure 5B, Figure S3: subplots x1, y1, z1), this finding was not unexpected, as the majority of Glire species diverged earlier [∼ 70 million years ago (MYA)] than Primate species (∼ 40 MYA).

**Figure 6:**
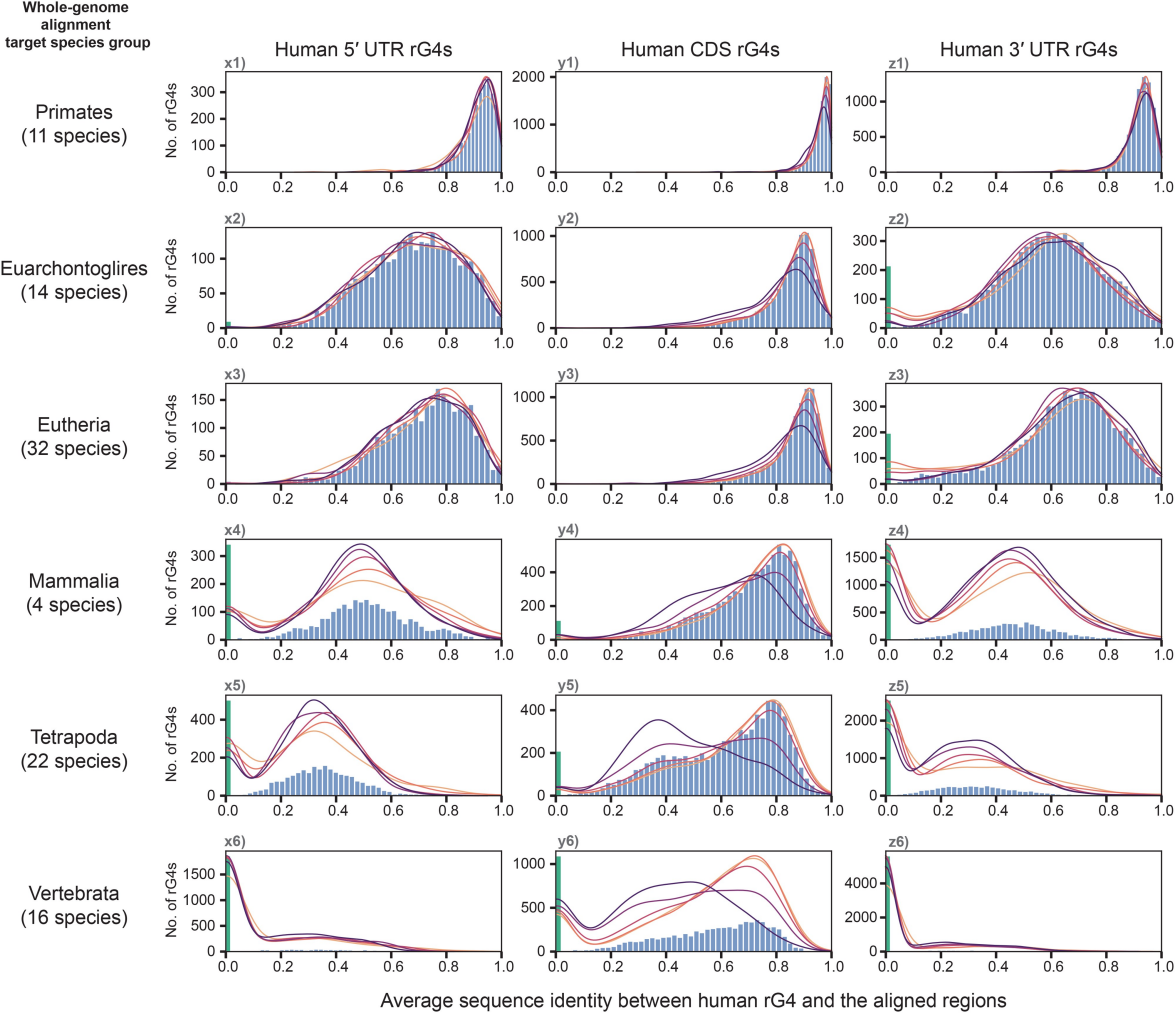
The divergence of the sequence of human rG4s across vertebrate species The evolutionary sequence divergence of human rG4s was determined using whole-genome multiple alignments. The sequence identities (using human rG4s as references) of the genomic alignments from all species were calculated and averaged to produce the average sequence identity metric. rG4s without proper genomic alignments in all species were assigned an average sequence identity of 0.0, and the corresponding bar plot is highlighted in green. Vertebrate species were further grouped into representative evolutionary clades (in reverse-chronological order) to illustrate the correlations between evolutionary times and sequence divergence. The member species of each clade and the estimated evolutionary divergence times are shown in Figure S1. rG4s were further grouped by their harboring gene regions (5′ UTR/CDS/3′ UTR) to highlight differences in their conservation statuses. The bar plot shows the overall distribution of the sequence divergence of all rG4s regardless of their canonicality. The overlaid kernel distribution estimation plot shows the distribution of individual rG4s of different canonicalities.

Furthermore, sequence identity analysis offered additional information that helped explain the observations from the PQS alignments. In humans and mice, the average sequence identities of the alignments were generally higher in CDS rG4s (**Figure 6**, Figure S4: subplots y1–6) than in UTR rG4s (Figure 6, Figure S4: subplots x1–6, z1–6), and higher in more recently diverged categories (Figure 6, Figure S4: subplots x1–3, y1–3, z1–3) than in those that diverged earlier (Figure 6, Figure S4: subplots x4–6, y4–6, z4–6), suggesting that the rG4 sequences had mutated at different rates depending on their gene regions. A closer inspection of the associations between sequence identities and rG4 canonicality of CDS rG4s (Figure S5) revealed that, in general, rG4s with higher canonicality exhibit insignificant differences in sequence identity, whereas rG4s with lower canonicality tend to be associated with lower sequence identity. This pattern is consistent between human and mouse observations and is more pronounced in earlier diverged species groups compared to recently diverged groups.

Meanwhile, the number of rG4s with an average sequence identity of 0, indicating that no alignments were found among all species, was shown to increase drastically among the early-diverged categories (Figure 6, Figure S4: leftmost green bar in the subplots). The absence of alignment suggests that the ancestral sequences of many modern human and mouse rG4s might not be present in older vertebrate genomes and were only introduced more recently in vertebrate evolution.

In line with previous findings on the distribution and conservation of rG4s, the evidence outlines a landscape wherein most rG4s in humans and mice were introduced relatively recently, shedding light on why the positional conservation of rG4s across the long-term eukaryotic evolution generally has been poor. The drastic changes in PQS alignment and the evolutionary sequence identity of UTR rG4s between the Eutheria and Mammalia categories suggest that a significant evolutionary event that drove the introduction of rG4s may have occurred during mammalian evolution. In contrast, the introduction of CDS rG4s appeared to have occurred gradually throughout vertebrate evolution.

### Sequence mutations might have different effects on the evolution of CDS rG4s and UTR rG4s

**Figure 7:**
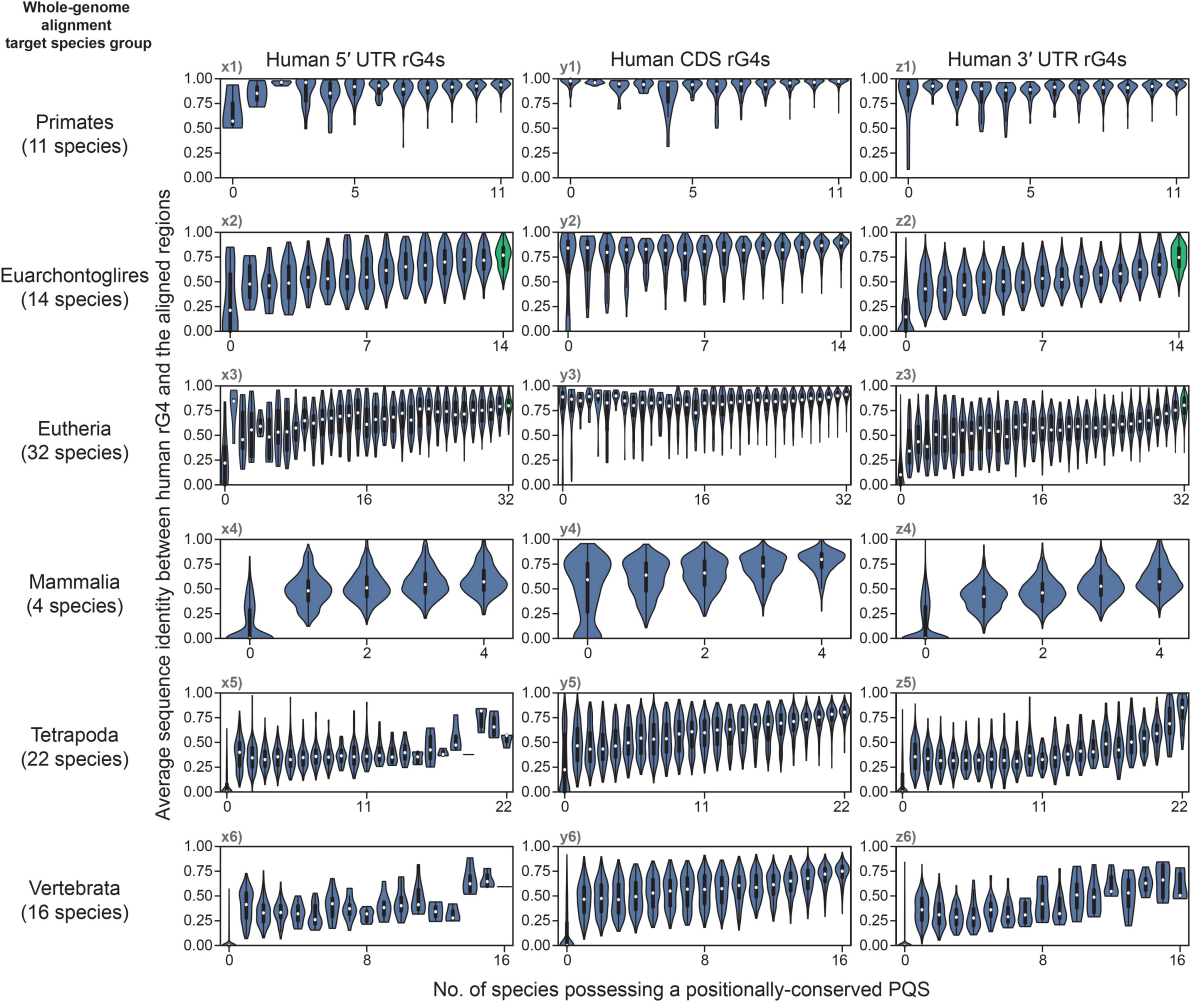
The associations between PQS conservation and the sequence identities of human rG4s Information from Figures 5 and 6 is combined here to illustrate the association between PQS conservation and the sequence divergence of human rG4s. Individual violin plots show the distribution of average sequence identities (from Figure 6); the rG4s associated with different PQS conservation statuses (Figure 5) are shown in different violin plots. Vertebrate species were further grouped into representative evolutionary clades (in reverse chronological order) to illustrate the correlations between evolutionary times and rG4 evolution. rG4s were further grouped by their harboring gene regions (5′ UTR/CDS/3′ UTR) to highlight differences in their conservation status. Under normal circumstances, higher PQS conservation might correlate with lower sequence divergence. However, this type of correlation is not observed in some subplots (*e.g*., x1, y1, z1, y2, y3), suggesting that under specific circumstances (*e.g*., gene region and evolutionary times), sequence divergence and PQS conservation may not influence each other.

When considering only more recent mammalian evolution in the Eutheria, Euarchontoglires, Primates, and Glires categories, the association between evolutionary sequence identity and PQS conservation was found to differ between CDS and UTR rG4s (**Figure 7**, Figure S6: subplots x1–3, y1–3, z1–3). For CDS rG4s, the sequence identity was roughly similar for all human and mouse rG4s, regardless of the number of species harboring a conserved PQS (Figure 7, Figure S6: subplots y1–3). This finding suggests conservation status of PQS is not apparently correlated with sequence identity, thus high evolutionary sequence identity may not be sufficient for PQS conservation. Regardless of their PQS conservation status, all rG4s seem to have experienced similar rates of mutation during evolution, but the differences in these mutations led to distinct outcomes in terms of PQS conservation in each species. In contrast, UTR rG4s experienced a more diverse rate of mutation, and a high number of mutations appeared to be associated with the loss of PQS in the aligned species (Figure 7, Figure S6: subplots x1–3, z1–3). Nevertheless, the median sequence identity was only about 0.7–0.75, even for UTR rG4s corresponding to PQSs conserved among all species in the Euarchontoglires or Eutheria categories (Figure 7, Figure S6: violin plots highlighted in green). This observation suggests that high sequence identity might not be necessary for PQS conservation in the UTR regions.

### Comparisons with ancestral quadruplex sequences suggest distinct trends in the gains and losses of canonicality between UTR and CDS rG4s

Interestingly, in quadruplex sequence evolution, some patterns appeared to be associated with the canonicality of rG4s. For 3′ UTR rG4s, PQS conservation was generally poorer among rG4s with lower canonicality (Figure 5B, Figure S3: subplots z1–4). In comparison, the canonicality of CDS rG4s seemed to be less closely related to the PQS conservation status (Figure 5B, Figure S3: subplots y1–6). Instead, among CDS rG4s, higher canonicality was associated with lower sequence identity in general, and this was consistent across all six species groups (Figure 7, Figure S6, subplots y1–6). While these findings suggest that the patterns of canonicality and evolution are linked, it remains unclear how the canonicality of the quadruplex sequences has changed throughout evolution.

**Figure 8:**
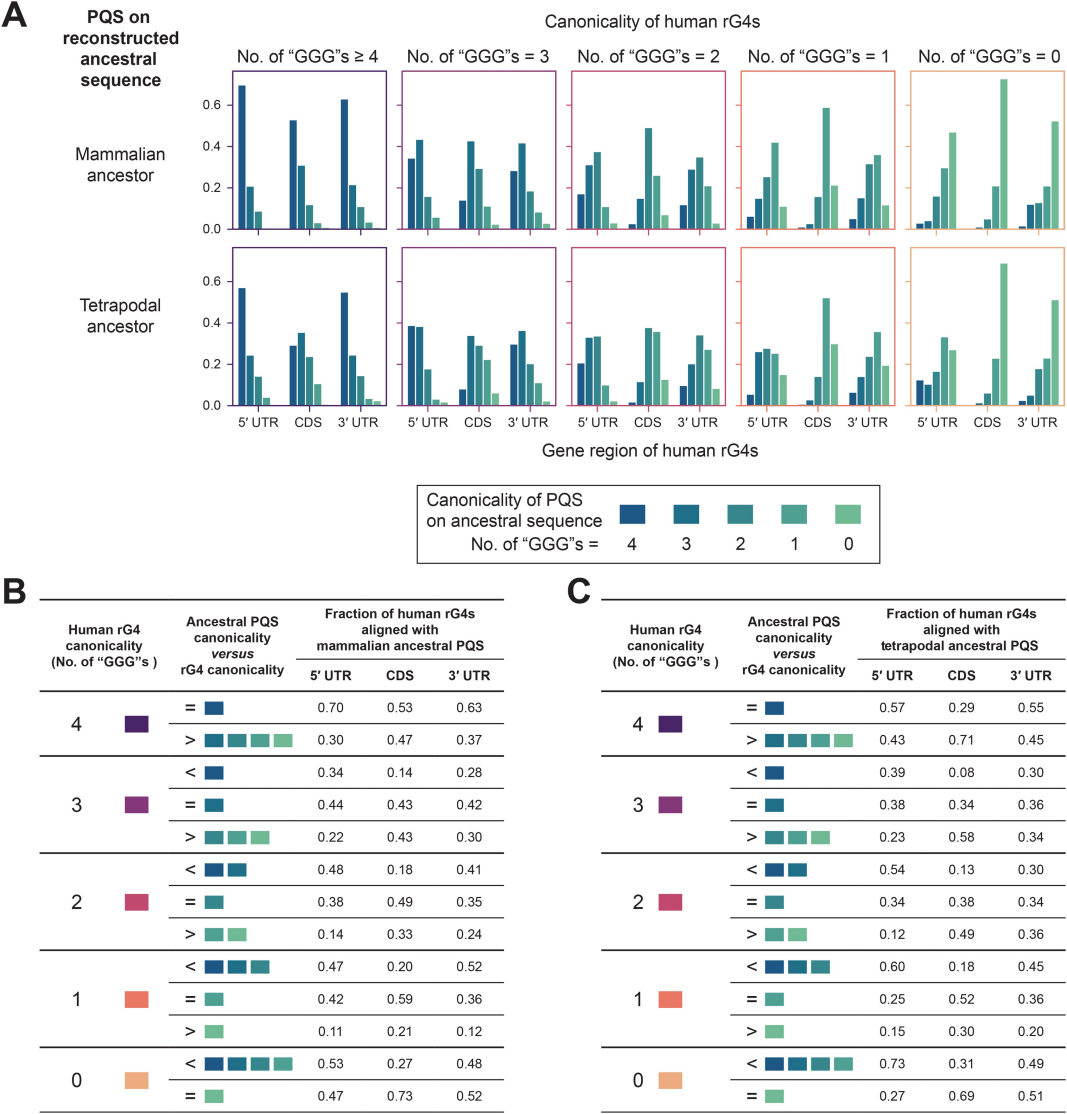
Comparison of the canonicality of human rG4s and the putative quadruplex sequences on ancestral sequences The ancestral sequences of human rG4s in the mammalian and tetrapodal clades were reconstructed from multiple alignments of genomic sequences. A PQS search of the ancestral sequences was performed, and the canonicality of the PQSs was determined as described in Figure 5A. The plot does not include ancestral sequences that lack a PQS. **A**. Landscape of canonicality in reconstructed mammalian and tetrapodal ancestral PQSs, classified by the canonicality of their aligned human rG4s. **B**. and **C**. Comparison of canonicality between human rG4s and their aligned mammalian (B) and tetrapodal (C) ancestral PQSs.

To further explore the evolution of quadruplex sequence canonicality, the ancestral sequences of human and mouse rG4s were reconstructed for the Mammalia and Tetrapoda clade, representing the period during which the most drastic change in the rG4 landscape seems to have occurred. The canonicality of the PQSs on the ancestral sequences (*i.e.*, ancestral PQSs) was compared with that of the human/mouse rG4s to evaluate the changes in quadruplex sequence canonicality during evolution. Changes in canonicality were found to occur frequently during evolution (**Figure 8**A, Figure S7A), and the canonicality of modern-date CDS rG4s tended to be higher than that of the ancestral PQSs (Figure 8B and C, Figure S7B and C). However, the canonicality of modern-date UTR rG4s tended to be lower than that of the ancestral PQSs (Figure 8B and C, Figure S7B and C). Such trends were especially apparent in some cases: CDS rG4s had a canonicality of 4, whereas most tetrapodal ancestral PQSs had a canonicality of less than 4 (Figure 8C, Figure S7C), suggesting that these motifs gained additional (GGG) G-tracts during evolution. The opposite was observed for UTR rG4s, which had a canonicality of 0, and most of the Mammalian and tetrapodal ancestral PQSs had a canonicality greater than 0 (Figure 8B and C, Figure S7B and C), suggesting that these motifs had degenerated and lost (GGG) G-tracts during evolution. These changes in canonicality suggest that CDS and UTR rG4s have distinct modes of evolution: in general, CDS rG4s gained canonicality and UTR rG4s lost canonicality during evolution.

### A cross-platform database application for accessing rG4 detections

In this study, we selected widely used cell and animal models, including ENCODE cell lines such as K562 and Bruce4 ES, for our rG4-seq experiments. Through our choice of models, we aimed to enhance compatibility and enable the reuse of the databases generated using these models. Furthermore, we have implemented a database application, the rG4-seq Database, to ensure that the curated rG4 detection results produced in this study are available to other researchers. The database program is available for the Windows, Linux, and MacOS platforms and offers a graphical interface for listing and searching for rG4s (Figure S8A); browsing rG4s in a genome-browser interface (Figure S8B); and examining the detailed information on each rG4 (Figure S8C).

The database interface was designed to ensure user-friendliness for non-bioinformatics specialists, especially those who wish to explore rG4s in a particular gene of interest and in non-human model species. We believe that the improved availability of and convenient access to the data will help researchers elaborate on the concept of NC-rG4s and encourage their inclusion as subjects in future functional explorations.

## Discussion

Historically, canonical rG4s residing on UTRs have been considered “low-hanging fruit” and thus have been the preferred subjects of rG4 research. Besides their accessibility with respect to computational prediction and experimental manipulation, the assumption that rG4s exert regulatory functions is well aligned with the widely accepted regulatory nature of UTRs [7,9,10,29,32]. In contrast, until recently, non-canonical rG4s and those residing in CDSs generally have received less attention because they are more challenging to discover and characterize [7,9,10,29,32]. We observed that the advancement of rG4 research is dependent on the reliable detection of new rG4 structures and identifying those that carry functional significance. Accordingly, this high-throughput investigation of rG4s in multiple model species follows this research paradigm to discover additional rG4s, identify conserved rG4s that could be functionally important, and support future exploration by providing a database application for other researchers to screen for suitable candidate rG4s.

Unfortunately, although the findings from this study confirm the presence of rG4s across the transcriptomes of various eukaryotes, evidence suggests an almost complete absence of positional conservation of CDS and UTR rG4s in long-term eukaryotic evolution, consistent with previous findings based on predicted rG4s [26]. In addition, the evolution of CDS rG4s was shown to be intertwined with the underlying AA sequences, and most UTR rG4s were found to have a relatively young evolutionary age. Such observations are not very encouraging, given that the investigation of better-understood RNA regulatory elements, such as microRNAs and splicing signals, has depended heavily on the conservation status of the elements. The observed lack of long-term conservation among rG4s will inevitably present challenges to further investigations of these RNA structures.

Nevertheless, the findings support an alternate hypothesis, namely that in eukaryotes, rG4s are inserted into the transcriptome substantially later than gene birth. It is also interesting to note that older genes and duplicated genes are more likely to have acquired rG4s than other genes, suggesting that the evolutionary process of introducing rG4s might involve selective pressure. Such an expansion would also involve changing the codon usage landscape and incorporating new quadruplex sequences into the UTR. This analysis provides an overview of the origins of the modern human and mouse rG4omes, which comprise two components with distinct origins: CDS rG4s accumulated gradually throughout evolution over a long-time scale, while UTR rG4s were introduced recently in mammalian evolution. Moreover, the underlying sequences of current rG4s seem to have continued to evolve over the last 100 MYA, such that the canonicality of the rG4s has continued to change.

Furthermore, a comparison of mouse and human rG4 sequences suggested that while many PQSs are positionally conserved between the two species, the rG4 structures were substantially less well conserved. Based on the analysis, one possible hypothesis to explain this phenomenon is as follows: a large, shared pool of rG4s was introduced into a common mammalian ancestor, after which gradual structural degeneration led to different sets of rG4s in different branches of mammals. In this light, some PQSs observed in the transcriptome would be the remnants of degenerated rG4s that still possess the G-tract and loop motif but have lost the ability to fold into a quadruplex. However, testing this hypothesis further would necessitate resolving the rG4 folding states of conserved PQSs across the tree of life of mammals; this would require either experimental characterization of the rG4omes of additional species or an improved *in silico* rG4 prediction algorithm.

The major limitation of the current study lies in the relatively small number of model species surveyed using rG4-seq. However, it is important to emphasize that our *in silico* evolutionary analysis further incorporated genomic data from nearly 100 additional species, enabling the resolution of rG4 and PQS positional conservation. This broader context was crucial for outlining the long-term evolutionary landscape of rG4s. At the same time, we carefully selected organisms for rG4-seq profiling, based on their status as representative models with rich genomic and transcriptomic resources. For instance, K562, HeLa, and Bruce4 cell lines are extensively profiled by the ENCODE project, while *Caenorhabditis elegans* and *Drosophila melanogaster* are part of the modENCODE project. The availability of comprehensive RNA-seq, structural, and regulatory datasets in these models facilitates future reuse of our rG4 datasets in functional studies.

Finally, resolving the recent evolutionary relationships between human and mouse rG4omes required whole-genome alignments, highlighting that the timing of UTR rG4 emergence in mammals could not be estimated accurately. Given that the observed rapid changes in UTR rG4 sequences were detected between the Eutheria clade (∼ 99 MYA) and the Mammalian clade (∼ 180 MYA), we propose two possible directions for future follow-up studies: (1) rG4s can be surveyed in model species from the Monotreme and Marsupial categories; these non-placental mammals are evolutionary outgroups in the Eutheria clade [33]. Evaluating the conservation of rG4s between the Monotreme, Marsupial, and Eutheria categories could enable a more accurate estimate of the origins of modern human UTR rG4s. (2) The period of rG4 changes overlaps with the Cretaceous Terrestrial Revolution (KTR), which featured intense diversification of angiosperms, insects, reptiles, birds, and mammals during the Middle to Late Cretaceous (125–80 MYA) [34,35]. The KTR provides a hypothesis to explain the difference in rG4ome size between fruit fly and zebrafish. In contrast to the fruit fly, zebrafish is not a land animal and was not likely to have been affected by the KTR; accordingly, the sizes of rG4omes might be linked to the KTR. Furthermore, angiosperm plant models have also been suggested to harbor many rG4s, although the studies did not use rG4-seq [4]. Future study could survey additional model angiosperm, reptile, and bird species. Although these studies may not identify novel rG4s conserved between humans and other model species, the results could elucidate the conditions and influences of evolution on changes in rG4ome sizes.

## Materials and methods

### rG4-seq datasets

For this study, previously published rG4-sequencing (rG4-seq) datasets from *Homo sapiens* HeLa cells and *Plasmodium falciparum* isolate 3D7 [3,19] were used.

### Total RNA sample preparation

#### *H. sapiens* K562 cell line

K562 cells were cultured according to the ENCODE protocol [36]. The cells were harvested and lysed using TRIzol reagent (Invitrogen, Waltham, MA). After chloroform extraction, total RNA was extracted from the aqueous supernatant using a RNeasy Kit (Qiagen, Hilden, Germany) according to the manufacturer’s protocol. DNase I digestion of each sample was performed to eliminate residual genomic DNA.

#### *Mus musculus* Bruce 4 ES cell line

*M. musculus* Bruce4 ES cells were cultured in T-175 flasks coated with a mouse embryonic fibroblast (MEF) feeder layer in a serum-containing ES medium. The embryonic stem (ES) medium comprised KnockOut Dulbecco’s modified Eagle’s medium (Gibco, Waltham, MA) supplemented with 15% (v/v) heat-inactivated fetal bovine serum (FBS; Hyclone, Logan, UT), 1× GlutaMax-I (Gibco), 1× non-essential amino acids (AAs; Gibco), 100 units (U)/ml penicillin/streptomycin (Gibco), 1× β-mercaptoethanol (Sigma, St. Louis, MO), and 10^6^ U/l leukemia inhibitory factor. TRIzol (Invitrogen) was used to extract RNA from harvested Bruce4 ES cells.

#### *Danio rerio*, whole organism

Zebrafish (*D. rerio*) employed in this experiment were generally provided by Michael Hon-Wah Lam from the City University of Hong Kong, China. Zebrafish were reared in charcoal-dechlorinated tap water under constant temperature conditions (28°C) with a light:dark cycle of 14:10  h. Fish were anesthetized in ice-cold aquarium water for 30 s, removed, and euthanized for RNA extraction by TRIzol reagent (Invitrogen).

#### *D. melanogaster* S2 cells

*D. melanogaster* S2 cells were cultured at 23°C in 20 ml of serum-containing medium per 250-ml filter-cap cell culture flasks. The medium comprised Schneider’s medium (Invitrogen) supplemented with 0.1 mg/ml penicillin/streptomycin (Invitrogen) and 10% (v/v) FBS (Invitrogen). After harvesting, the cells were lysed using TRIzol. After chloroform extraction, total RNA was extracted from the aqueous supernatant using a RNeasy Kit (Qiagen) according to the manufacturer’s protocol. DNase I digestion of each sample was performed to eliminate residual genomic DNA.

#### *C. elegans* isolate N2, mixed stage

*C. elegans* were maintained on nematode growth medium-containing agar plates seeded with a liquid culture of *Escherichia coli* strain OP50. To grow large quantities of *C. elegans* in liquid medium prior to RNA extraction, four large culture plates of *C. elegans* were added to a 1-l flask containing 250 ml of S medium inoculated with *E. coli* OP50 cells pelleted from 2 l of overnight culture. *C. elegans* were cultured for 4 days at 20°C and 150 r/min before harvesting.

Harvested mixed-stage *C. elegans* were washed with M9 buffer to remove *E. coli*, lysed using TRIzol, and freeze-thawed in liquid nitrogen [37]. After chloroform extraction, total RNA was extracted from the aqueous supernatant using a RNeasy Kit (Qiagen) according to the manufacturer’s protocol. DNase I digestion was performed to eliminate residual genomic DNA.

#### *Saccharomyces cerevisiae* strain BY4741

*S. cerevisiae* BY4741 was maintained on YPD agar plates. To culture a large quantity of *S. cerevisiae* for RNA extraction, a single colony was inoculated in 250 ml of YPD broth and cultured overnight at 30°C and 250 r/min before harvesting.

After harvesting and centrifugation, the *S. cerevisiae* pellet was resuspended in TRIzol and lysed by vortexing with 0.5-mm glass beads. After chloroform extraction, total RNA was extracted from the aqueous supernatant using a RNeasy Kit (Qiagen) according to the manufacturer’s protocol. DNase I digestion was performed to eliminate residual genomic DNA.

### rG4-seq experiments

The optimized protocol for the rG4-seq experiment was performed as previously described [38]. Briefly, total RNA was subjected to polyadenylated (polyA)-RNA selection (Invitrogen) followed by alkaline fragmentation to generate RNA fragments of approximately 250 nt in length. Purified fragmented RNAs were dephosphorylated with T4 PNK (NEB, Ipswich, MA) and rSAP (NEB) at 37°C for 30 min. Dephosphorylated RNAs were then ligated with 3′ adapter (5′-­/5rApp/NNNNNNAGATCGGAAGAGCACACGTCTG/3SpC3/-­3′) at 25°C for 1 h by T4 RNA ligase 2, truncated KQ ligase (NEB). The unused 3′ adapters were digested with 5′ deadenylase (NEB) and RecJf (NEB) and removed by column purification (Zymo Research, Irvine, CA) for reverse transcription (RT). Three conditions, including 150 mM K^+^, 150 mM Li^+^, and a condition with 2 µM PDS in the presence of 150 mM K^+^ (K^+^/PDS), were applied to RT reaction using SuperScript III Reverse Transcriptase (Invitrogen) with RT primer (5′-CAGACGTGTGCTCTTCCGATCT-3′) for the extension. The RT program in detail was followed as described in the previous reference [3]. Purified cDNAs were then ligated with a 5′ adapter (5′-/5Phos/AGATCGGAAGAGCGTCG TGTAGCTCTTCCGATCTN10/3SpC3/-3′) using Quick Ligation Kit (NEB) at 37°C overnight. The ligated products were then denatured and loaded on the 10% denaturing urea-TBE acrylamide gel (Invitrogen) for size selection. A 100–400 nt region of each sample was then excised and purified with RNA Clean & Concentrator^TM^ (Zymo Research). Purified products were then amplified using the forward primer (5′-/5Phos/AGATCGGAAGAGCGTCGTGTAGCTCTTCCGATCTN10/3SpC3/-3′) and the reverse primer (5′-CAAGCAGAAGACGGCATACGAGAT-(6 nt index_seq)-GTGACTGGAGTTCAGACGTGTGCTCTTCCGATCT-3′) with optimal cycles by KAPA HiFi HotStart ReadyMix (Roche, Basel, Switzerland). The PCR samples were resolved on the 1.8% TAE agarose gel, and the fragments ranging from 150 to 400 nt in size were cut out and extracted using gel purification (Zymo Research). The DNA libraries were quantified, pooled, and subsequently sequenced on the Illumina HiSeq System.

### rG4-seq data processing

Pre-processing and short read alignment of sequencing data were conducted as previously described using the *rG4-seeker* pipeline [18]. Sequencing reads were first adapter-trimmed and quality-trimmed using cutadapt [39]. Trimmed reads were then aligned to respective reference genome using STAR aligner [40], and only uniquely aligned reads were used for subsequent analysis. The rG4-seeker pipeline [18] was used for counting the read coverage, and number of read starts per nucleotide position, reverse transcriptase stalling (RTS) site analysis, and rG4 calling. The definition of rG4 structural motifs has been previously described in [3]. Sequencing reads from rG4-stabilizing conditions (K^+^ and K^+^/PDS) were compared to those from non-rG4-stabilizing conditions (Li^+^) to detect RTS sites specific to the rG4-stabilizing conditions, using a target false discovery rate (FDR) of 0.05. The comparison resulted in four sets of RTS sites detections (two replicates, two rG4-stabilizing conditions) in two-replicate rG4-seq experiments (all samples except human HeLa), and eight sets of RTS sites detections in four-replicate rG4-seq experiments (human HeLa sample). Each set of detected RTS sites was then analysed independently to identify the rG4 motifs responsible for generating the RTS event. Finally, rG4s detected in two or more detection sets were considered to be genuine and included in the species-specific rG4 structome.

The human rG4 structome was obtained by first conducting rG4 calling using rG4-seeker independently for K562 and HeLa cell samples, then taking the union of the detections and combining the identical underlying rG4 motif sequences to avoid duplicates. The intersection and union of identified rG4s across the detection sets and samples were computed based on the overlapping of genomic coordinates of the rG4 sequences. The *Plasmodium* rG4 detection results from [19] were reused in this study.

Detected rG4s were first assigned structural motifs based on their motif sequences, which were then used for further grouping into motif classes (G3, G2, variant G2) (Figure 1D). Only G3 and G2 rG4s were utilized in subsequent analyses, while variant G2 rG4s were excluded. All detected rG4s were reported in the rG4-seq database.

### Bioinformatic analysis

#### Transcriptome and peptide sequence annotations

The transcriptome annotations were downloaded from Ensembl (v101) [41]. The matched protein-CDSs and the peptide sequences of the canonical isoforms of protein-coding genes were downloaded from UCSC [31] and Ensembl (v101), respectively. For downstream analysis at the peptide sequence level, only isoforms with identical CDSs and peptide sequences in both databases were selected to ensure coherence.

#### Determining rG4 overlapping with 3′ UTR microRNA binding site

TargetScan default 3′ UTR microRNA binding sites predictions and UTR sequences for human (v8.0), mouse (v8.0), and fruit fly (v7.2) were downloaded from the TargetScan homepage [30]. The transcript coordinates of fruit fly predictions were converted to genomic coordinates by matching the provided UTR sequences with the Ensembl transcriptome annotations. The genomic coordinates of human predictions were converted from hg19 to hg38 using LiftOver [31]. A microRNA binding site is considered overlapping with a rG4 if the seed region is located within ±10 nt of the rG4 motif sequence.

#### DNA REs and peptide LCR analysis

The annotations for REs were downloaded from UCSC. Overlapping between rG4s and DNA REs was determined using the respective genomic coordinates. Peptide LCR analysis was performed as previously described [19].

#### Amino acid residue and codon composition analysis

The locations of CDS rG4s on peptide sequences were determined by first identifying the coordinates of each rG4 on its parent CDS sequence, then translating the CDS coordinates to the peptide coordinates according to the reading frame.

#### Mapping evolutionary ages to conserved protein-coding gene families

After obtaining the complete list of species included in the Ensembl (v101) database, a species tree was constructed using evolutionary age information from TimeTree [42]. The gene trees for all conserved protein-coding gene families were downloaded from Ensembl. The list of species in each gene tree was extracted to identify the last common ancestor in the species tree. The evolutionary age of the common ancestor was assigned as the age of the gene family.

#### Determining the whole-genome duplication status of genes

The list of human and mouse genes retained from two rounds of whole-genome duplication (2R-WGD) in vertebrate evolution (Ohnologs) was downloaded from the OHNOLOGS v2 database using the “Relaxed” criterion [43].

#### PQS identification

PQSs from nucleotide sequences were identified using the PQS prediction module implemented in *rG4-seeker*.

#### rG4 positional conservation analysis

For the position conservation analysis of CDS rG4s, multiple alignments of CDSs with respect to the translated peptide sequences were conducted using MACSE [44] and MAFFT [45]. Multiple alignments were performed for each conserved protein-coding gene family listed in Ensembl (v101), using all protein-coding isoforms. Two CDS rG4s from different species were considered positionally conserved if their multiple alignment outcomes overlapped.

For the position conservation analysis comparing human and mouse UTR rG4s, the rG4s of one species were mapped to the genome of the other species using the UCSC LiftOver tool [31]. The conversion of genomic coordinates from human hg38 to mouse mm10 genome was conducted using the hg38ToMm10 chain file (https://hgdownload.soe.ucsc.edu/goldenPath/hg38/liftOver/hg38ToMm10.over.chain.gz), and the conversion from mouse mm10 genome to human hg38 genome was conducted using the mm10ToHg38 chain file (https://hgdownload.soe.ucsc.edu/goldenPath/mm10/liftOver/mm10ToHg38.over.chain.gz). A human UTR rG4 was considered positionally conserved if its genomic coordinates overlapped with any of the converted mouse UTR rG4s, and *vice versa* for the mouse UTR rG4s.

#### Conservation analysis of quadruplex sequences using whole-genome multiple alignment

The 100-way and 60-way vertebrate whole-genome multiple alignments for the human hg38 and mouse mm10 genomes were downloaded from UCSC [31]. The list of aligned species was used to construct a species tree, with evolutionary ages determined using information from TimeTree [42]. The species were regrouped into representative groups based on their evolutionary clades. Sense strand sequences from other species that were aligned with the human/mouse rG4 motifs plus 30 nt of upstream/downstream sequences were extracted for conservation analysis. A PQS search of the aligned sequences was performed using the sequence motif definitions (canonical/G_3_L_1-7_, long loop, bulges, and 2-quartet) described previously [3]. The sequence identity between the aligned and human/mouse sequences was calculated by dividing the number of matched nucleotides by the human/mouse sequence length.

#### Ancestral sequence reconstruction

Reconstruction of the ancestral sequences was conducted using the Python package TreeTime [46], supplemented with the UCSC whole-genome multiple alignment data and the phylogenetic tree with alignment distances. Ancestors in the class Mammalia and superclass Tetrapoda were reconstructed using only species that belonged to the respective clades.

### rG4-seq database application

The graphical user interface of the database application was implemented in Python using the PySide6 framework. The igv.js [47] was used to implement the embedded genome browser interface. Standalone executables designed for the Windows, Linux, and MacOS platforms were compiled using Nuitka. The database backend application was implemented using NGINX and hosted at https://rg4s.science/, which is backed by a Linux server.

## Supplementary Material

qzaf078_Supplementary_Data

## Data Availability

The raw data from the rG4-seq experiments have been deposited in the Genome Sequence Archive [48] at the National Genomics Data Center (NGDC), China National Center for Bioinformation (CNCB) (GSA: PRJCA032791 and GSA-Human: PRJCA033501), and are publicly accessible at https://ngdc.cncb.ac.cn/gsa. The raw data from the rG4-seq experiments are also available at the Sequence Read Archive (SRA: PRJNA1071355). The rG4-seq Database program is available on the https://rg4s.science portal website.

## CRediT author statement

**Eugene Yui-Ching Chow:** Methodology, Investigation, Software, Formal analysis, Data curation, Visualization, Writing – original draft. **Jieyu Zhao:** Methodology, Investigation, Writing – original draft. **Chun Kit Kwok:** Conceptualization, Resources, Supervision, Project administration, Writing – review & editing, Funding acquisition. **Ting Fung Chan:** Conceptualization, Resources, Supervision, Project administration, Writing – review & editing, Funding acquisition. All authors have read and approved the final manuscript.
