# Supplementary material for: Differential Evolution of CDS and UTR Non-canonical RNA G-quadruplex Structures in Eukaryotic Transcriptomes
Source: Genomics Proteomics Bioinformatics. 2025 Sep 14;23(6):qzaf078. doi: 10.1093/gpbjnl/qzaf078 (PMC13198871; doi:10.1093/gpbjnl/qzaf078)
Supplement: qzaf078_Supplementary_Data [file qzaf078_supplementary_data.zip › table_S3.docx]

**Table S3 Statistics of rG4s overlapping with splice junctions**

| **rG4 structural motifs** | **No. of rG4s overlapping splice junctions** | **Percentage** |
| --- | --- | --- |
| Human |  |  |
| G3 |  |  |
| Canonical/G3L1-7 | 26 | 1.55% |
| Long loop | 31 | 2.16% |
| G2 |  |  |
| Bulges | 336 | 5.45% |
| Two-quartet | 943 | 8.53% |
| Mouse |  |  |
| G3 |  |  |
| Canonical/G3L1-7 | 6 | 0.50% |
| Long loop | 16 | 2.01% |
| G2 |  |  |
| Bulges | 235 | 5.49% |
| Two-quartet | 769 | 8.29% |
| Zebrafish |  |  |
| G3 |  |  |
| Canonical/G3L1-7 | 2 | 3.51% |
| Long loop | 5 | 9.43% |
| G2 |  |  |
| Bulges | 41 | 11.61% |
| Two-quartet | 90 | 10.30% |
| Fruit fly |  |  |
| G3 |  |  |
| Canonical/G3L1-7 | 1 | 3.33% |
| Long loop | 1 | 3.45% |
| G2 |  |  |
| Bulges | 14 | 1.59% |
| Two-quartet | 109 | 3.13% |
| Nematode |  |  |
| G3 |  |  |
| Canonical/G3L1-7 | 1 | 33.33% |
| Long loop | 0 | 0.00% |
| G2 |  |  |
| Bulges | 14 | 8.38% |
| Two-quartet | 109 | 8.00% |
| Yeast |  |  |
| G3 |  |  |
| Canonical/G3L1-7 | 0 | 0.00% |
| Long loop | 0 | 0.00% |
| G2 |  |  |
| Bulges | 0 | 0.00% |
| Two-quartet | 0 | 0.00% |
| *Plasmodium* |  |  |
| G3 |  |  |
| Canonical/G3L1-7 | 1 | 16.67% |
| Long loop | 0 | 0.00% |
| G2 |  |  |
| Bulges | 4 | 3.42% |
| Two-quartet | 25 | 4.11% |
