# Supplementary material for: Differential Evolution of CDS and UTR Non-canonical RNA G-quadruplex Structures in Eukaryotic Transcriptomes
Source: Genomics Proteomics Bioinformatics. 2025 Sep 14;23(6):qzaf078. doi: 10.1093/gpbjnl/qzaf078 (PMC13198871; doi:10.1093/gpbjnl/qzaf078)
Supplement: qzaf078_Supplementary_Data [file qzaf078_supplementary_data.zip › Figure_S1.pdf]

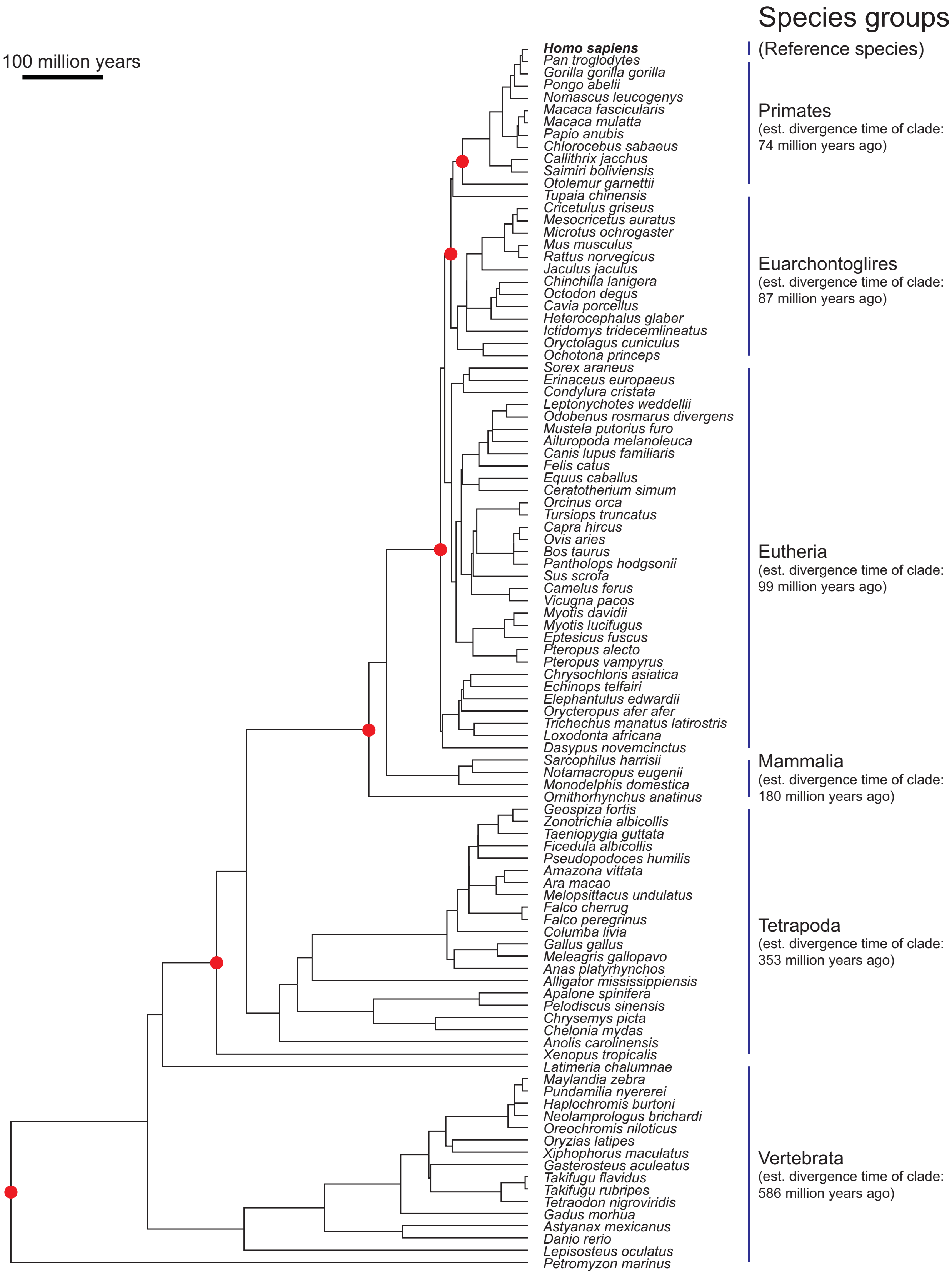

Species groups
100 million years
Homo sapiens
Pan troglodytes
Gorilla gorilla gorilla
Pongo abelii
Nomascus leucogenys
Macaca fascicularis
Macaca mulatta
Papio anubis
Chlorocebus sabaeus
Callithrix jacchus
Saimiri boliviensis
Otolemur garnettii
Tupaia chinensis
Cricetulus griseus
Mesocricetus auratus
Microtus ochrogaster
Mus musculus
Rattus norvegicus
Jaculus jaculus
Chinchilla lanigera
Octodon degus
Cavia porcellus
Heterocephalus glaber
Ictidomys tridecemlineatus
Oryctolagus cuniculus
Ochotona princeps
Sorex araneus
Erinaceus europaeus
Condylura cristata
Leptonychotes weddellii
Odobenus rosmarus divergens
Mustela putorius furo
Ailuropoda melanoleuca
Canis lupus familiaris
Felis catus
Equus caballus
Ceratotherium simum
Orcinus orca
Tursiops truncatus
Capra hircus
Ovis aries
Bos taurus
Pantholops hodgsonii
Sus scrofa
Camelus ferus
Vicugna pacos
Myotis davidii
Myotis lucifugus
Eptesicus fuscus
Pteropus alecto
Pteropus vampyrus
Chrysochloris asiatica
Echinops telfairi
Elephantulus edwardii
Orycteropus afer afer
Trichechus manatus latirostris
Loxodonta africana
Dasypus novemcinctus
Sarcophilus harrisii
Notamacropus eugenii
Monodelphis domestica
Ornithorhynchus anatinus
Geospiza fortis
Zonotrichia albicollis
Taeniopygia guttata
Ficedula albicollis
Pseudopodoces humilis
Amazona vittata
Ara macao
Melopsittacus undulatus
Falco cherrug
Falco peregrinus
Columba livia
Gallus gallus
Meleagris gallopavo
Anas platyrhynchos
Alligator mississippiensis
Apalone spinifera
Pelodiscus sinensis
Chrysemys picta
Chelonia mydas
Anolis carolinensis
Xenopus tropicalis
Latimeria chalumnae
Maylandia zebra
Pundamilia nyererei
Haplochromis burtoni
Neolamprologus brichardi
Oreochromis niloticus
Oryzias latipes
Xiphophorus maculatus
Gasterosteus aculeatus
Takifugu flavidus
Takifugu rubripes
Tetraodon nigroviridis
Gadus morhua
Astyanax mexicanus
Danio rerio
Lepisosteus oculatus
Petromyzon marinus
(Reference species)
Primates
(est. divergence time of clade: 74 million years ago)
Euarchontoglires
(est. divergence time of clade: 87 million years ago)
Eutheria
(est. divergence time of clade: 99 million years ago)
Mammalia
(est. divergence time of clade: 180 million years ago)
Tetrapoda
(est. divergence time of clade: 353 million years ago)
Vertebrata
(est. divergence time of clade: 586 million years ago)
