# Supplementary material for: Differential Evolution of CDS and UTR Non-canonical RNA G-quadruplex Structures in Eukaryotic Transcriptomes
Source: Genomics Proteomics Bioinformatics. 2025 Sep 14;23(6):qzaf078. doi: 10.1093/gpbjnl/qzaf078 (PMC13198871; doi:10.1093/gpbjnl/qzaf078)
Supplement: qzaf078_Supplementary_Data [file qzaf078_supplementary_data.zip › Figure_S2.pdf]

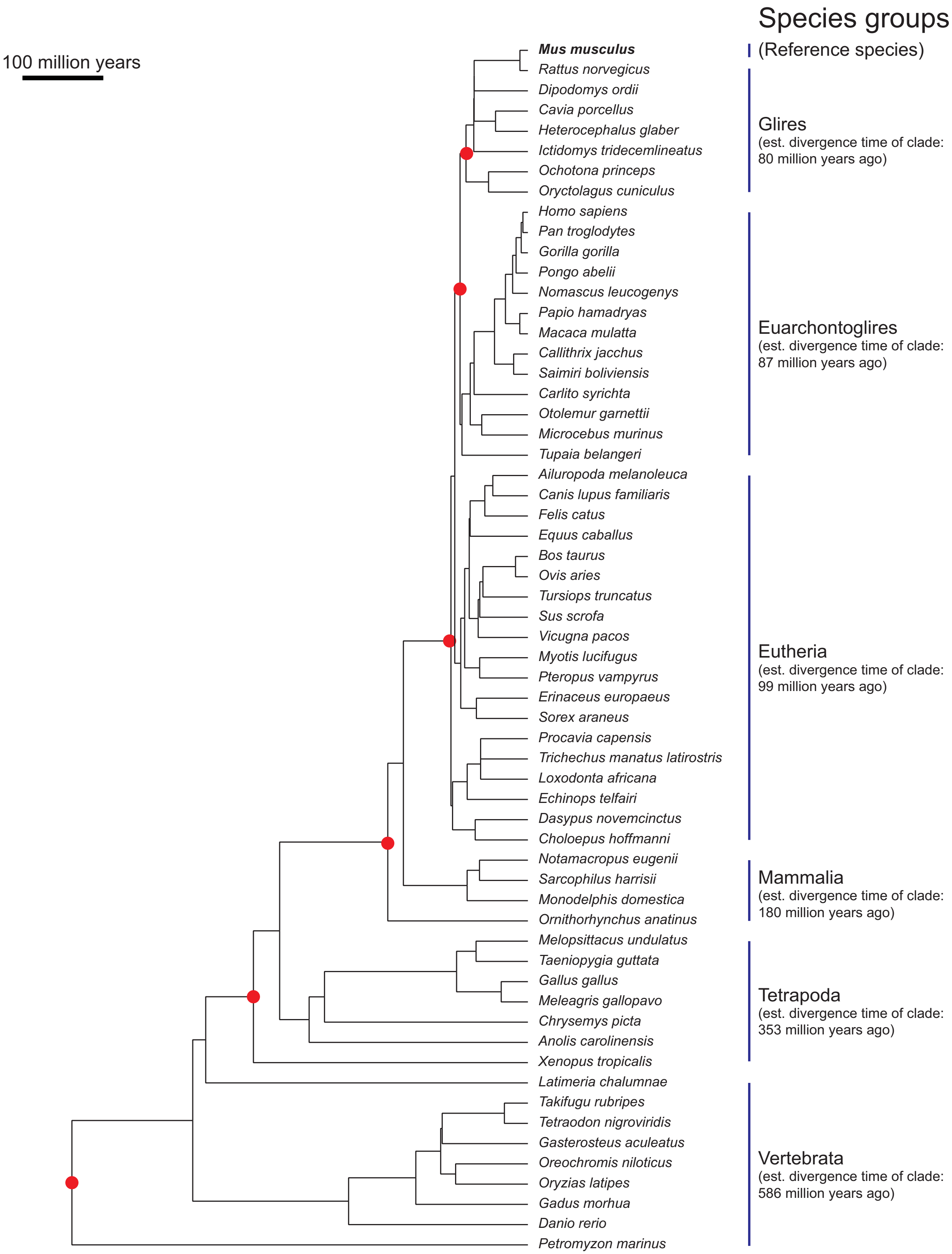
Species groups
100 million years
Mus musculus
Rattus norvegicus
Dipodomys ordii
Cavia porcellus
Heterocephalus glaber
Ictidomys tridecemlineatus
Ochotona princeps
Oryctolagus cuniculus
Homo sapiens
Pan troglodytes
Gorilla gorilla
Pongo abelii
Nomascus leucogenys
Papio hamadryas
Macaca mulatta
Callithrix jacchus
Saimiri boliviensis
Carlito syrichta
Otolemur garnettii
Microcebus murinus
Tupaia belangeri
Ailuropoda melanoleuca
Canis lupus familiaris
Felis catus
Equus caballus
Bos taurus
Ovis aries
Tursiops truncatus
Sus scrofa
Vicugna pacos
Myotis lucifugus
Pteropus vampyrus
Erinaceus europaeus
Sorex araneus
Procavia capensis
Trichechus manatus latirostris
Loxodonta africana
Echinops telfairi
Dasypus novemcinctus
Choloepus hoffmanni
Notamacropus eugenii
Sarcophilus harrisii
Monodelphis domestica
Ornithorhynchus anatinus
Melopsittacus undulatus
Taeniopygia guttata
Gallus gallus
Meleagris gallopavo
Chrysemys picta
Anolis carolinensis
Xenopus tropicalis
Latimeria chalumnae
Takifugu rubripes
Tetraodon nigroviridis
Gasterosteus aculeatus
Oreochromis niloticus
Oryzias latipes
Gadus morhua
Danio rerio
Petromyzon marinus
(Reference species)
Glires
(est. divergence time of clade: 80 million years ago)
Euarchontoglires
(est. divergence time of clade: 87 million years ago)
Eutheria
(est. divergence time of clade: 99 million years ago)
Mammalia
(est. divergence time of clade: 180 million years ago)
Tetrapoda
(est. divergence time of clade: 353 million years ago)
Vertebrata
(est. divergence time of clade: 586 million years ago)
