# Supplementary material for: Differential Evolution of CDS and UTR Non-canonical RNA G-quadruplex Structures in Eukaryotic Transcriptomes
Source: Genomics Proteomics Bioinformatics. 2025 Sep 14;23(6):qzaf078. doi: 10.1093/gpbjnl/qzaf078 (PMC13198871; doi:10.1093/gpbjnl/qzaf078)
Supplement: qzaf078_Supplementary_Data [file qzaf078_supplementary_data.zip › Figure_S4.pdf]

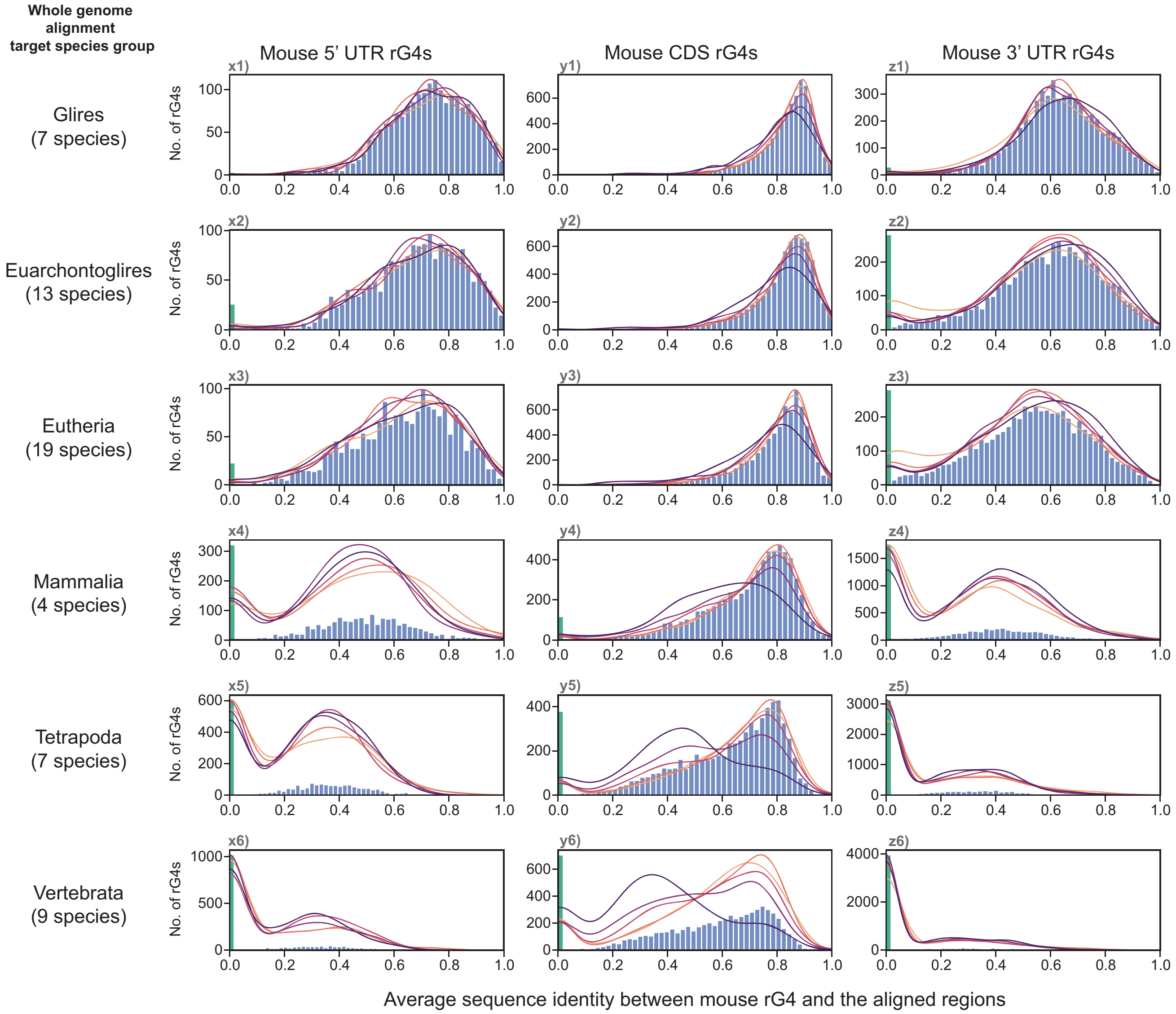
Whole genome alignment target species group
Mouse 5' UTR rG4s
Mouse CDS rG4s
Mouse 3' UTR rG4s
Glires (7 species)
Euarchontoglires (13 species)
Eutheria (19 species)
Mammalia (4 species)
Tetrapoda (7 species)
Vertebrata (9 species)
x1)
y1)
z1)
x2)
y2)
z2)
x3)
y3)
z3)
x4)
y4)
z4)
x5)
y5)
z5)
x6)
y6)
z6)
No. of rG4s
Average sequence identity between mouse rG4 and the aligned regions
