# Supplementary material for: Differential Evolution of CDS and UTR Non-canonical RNA G-quadruplex Structures in Eukaryotic Transcriptomes
Source: Genomics Proteomics Bioinformatics. 2025 Sep 14;23(6):qzaf078. doi: 10.1093/gpbjnl/qzaf078 (PMC13198871; doi:10.1093/gpbjnl/qzaf078)
Supplement: qzaf078_Supplementary_Data [file qzaf078_supplementary_data.zip › Figure_S7.pdf]

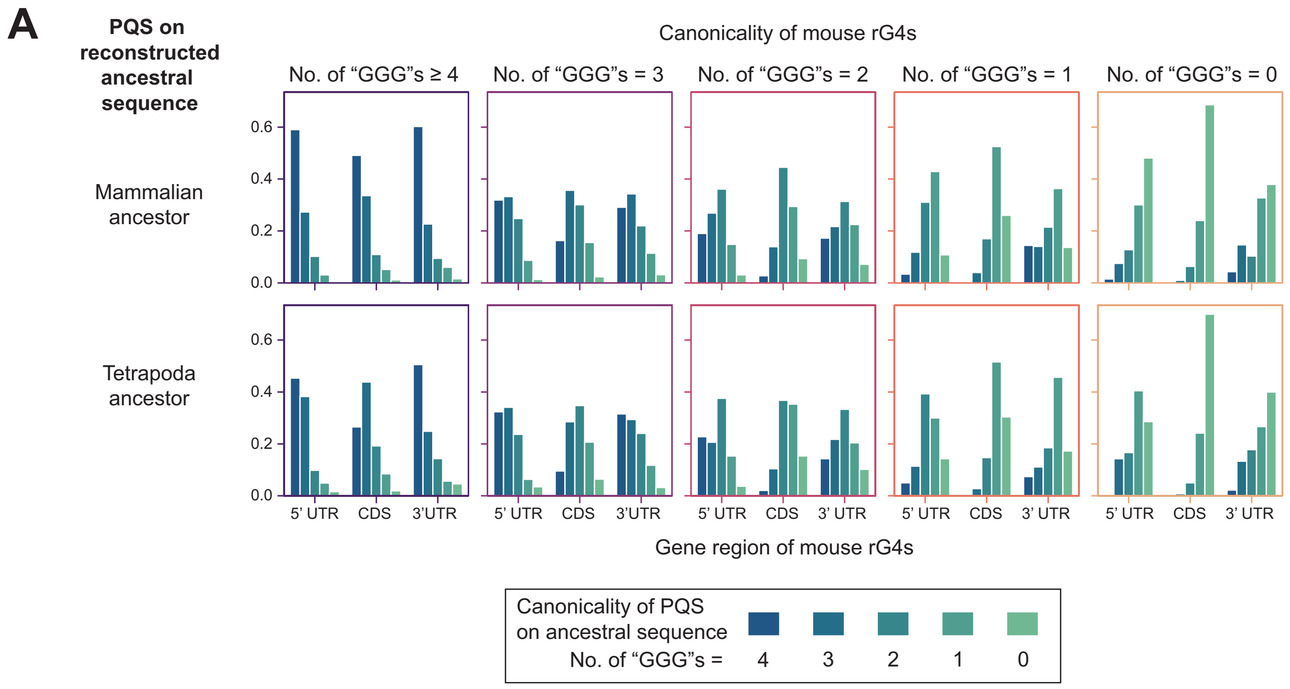

B

Mouse rG4 canonicality (No. of "GGG"s)	Ancestral PQS canonicality versus rG4 canonicality	Fraction of mouse rG4s aligned with Mammalia ancestral PQS		
		5' UTR	CDS	3' UTR
4	=	0.59	0.49	0.60
	>	0.41	0.51	0.40
3	<	0.32	0.16	0.29
	=	0.33	0.36	0.34
	>	0.35	0.48	0.37
2	<	0.46	0.17	0.39
	=	0.36	0.45	0.31
	>	0.18	0.39	0.30
1	<	0.46	0.21	0.50
	=	0.43	0.53	0.36
	>	0.11	0.26	0.14
0	<	0.52	0.31	0.62
	=	0.48	0.69	0.38

C

Mouse rG4 canonicality (No. of "GGG"s)	Ancestral PQS canonicality versus rG4 canonicality	Fraction of mouse rG4s aligned with Tetrapoda ancestral PQS		
		5' UTR	CDS	3' UTR
4	=	0.45	0.27	0.51
	>	0.55	0.73	0.49
3	<	0.32	0.10	0.32
	=	0.34	0.29	0.29
	>	0.34	0.62	0.39
2	<	0.43	0.13	0.36
	=	0.38	0.37	0.33
	>	0.19	0.51	0.31
1	<	0.56	0.18	0.37
	=	0.30	0.52	0.46
	>	0.14	0.30	0.17
0	<	0.71	0.30	0.60
	=	0.29	0.70	0.40
