# Supplementary material for: Differential Evolution of CDS and UTR Non-canonical RNA G-quadruplex Structures in Eukaryotic Transcriptomes
Source: Genomics Proteomics Bioinformatics. 2025 Sep 14;23(6):qzaf078. doi: 10.1093/gpbjnl/qzaf078 (PMC13198871; doi:10.1093/gpbjnl/qzaf078)
Supplement: qzaf078_Supplementary_Data [file qzaf078_supplementary_data.zip › Figure_S8.pdf]

A

B

C

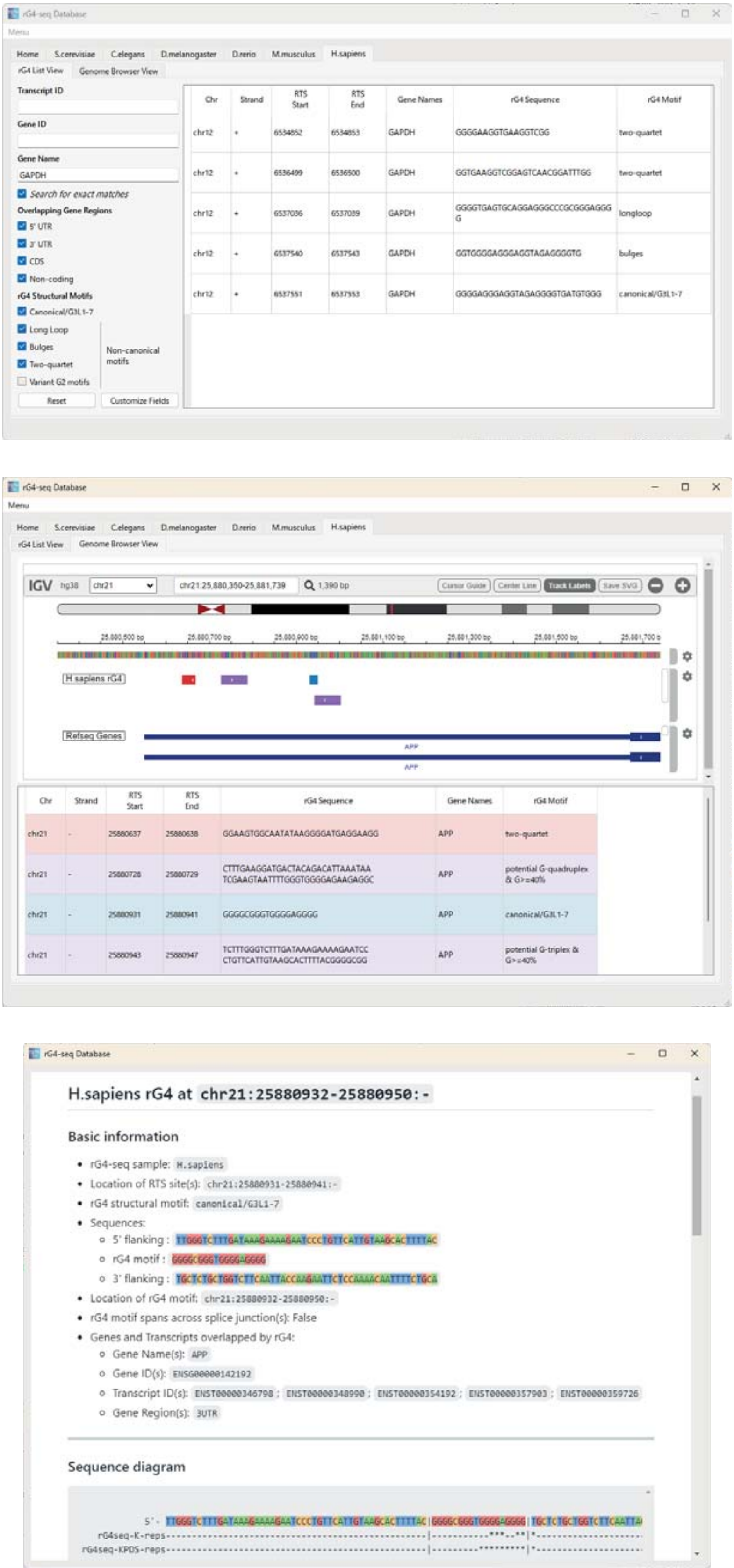

rG4-seq Database
Menu
Home
S.cerevisiae
C.elegans
D.melanogaster
D.rerio
M.musculus
H.sapiens
rG4 List View
Genome Browser View
Transcript ID
Gene ID
Gene Name
GAPDH
Search for exact matches
Overlapping Gene Regions
5' UTR
3' UTR
CDS
Non-coding
rG4 Structural Motifs
Canonical/G3L1-7
Long Loop
Bulges
Two-quartet
Variant G2 motifs
Non-canonical motifs
Reset
Customize Fields
Chr
Strand
RTS Start
RTS End
Gene Names
rG4 Sequence
rG4 Motif
two-quartet
longloop
bulges
canonical/G3L1-7
IGV
hg38
chr21
Cursor Guide
Center Line
Track Labels
Save SVG
H.sapiens rG4
Refseq Genes
APP
potential G-quadruplex & G>=40%
potential G-triplex & G>=40%
H.sapiens rG4 at chr21:25880932-25880950:-
Basic information
rG4-seq sample: H.sapiens
Location of RTS site(s): chr21:25880931-25880941:-
rG4 structural motif: canonical/G3L1-7
Sequences:
5' flanking :
rG4 motif :
3' flanking :
Location of rG4 motif: chr21:25880932-25880950:-
rG4 motif spans across splice junction(s): False
Genes and Transcripts overlapped by rG4:
Gene Name(s): APP
Gene ID(s): ENSG00000142192
Transcript ID(s): ENST00000346798 ; ENST00000348990 ; ENST00000354192 ; ENST00000357903 ; ENST00000359726
Gene Region(s): 3UTR
Sequence diagram
rG4seq-K-reps
rG4seq-KPDS-reps
