# Supplementary material for: Differential Evolution of CDS and UTR Non-canonical RNA G-quadruplex Structures in Eukaryotic Transcriptomes
Source: Genomics Proteomics Bioinformatics. 2025 Sep 14;23(6):qzaf078. doi: 10.1093/gpbjnl/qzaf078 (PMC13198871; doi:10.1093/gpbjnl/qzaf078)
Supplement: qzaf078_Supplementary_Data [file qzaf078_supplementary_data.zip › Supplementary material captions.docx]

**Supplementary material**

**File S1 List of rG4s overlapping with predicted 3′ UTR miRNA binding sites**

**File S2 List of rG4s overlapping with splice junctions**

**Figure S1 Phylogenetic tree of the 100 species included in whole-genome multiple alignments, with *Homo sapiens* as the reference species**

The species are grouped into representative evolutionary categories. Red dots denote the last common ancestor in each species category.

**Figure S2 Phylogenetic tree of the 60 species included in whole-genome multiple alignments, with *Mus musculus* as the reference species**

The species are grouped into representative evolutionary categories. Red dots denote the last common ancestor in each species category.

**Figure S3 Conservation of the underlying quadruplex sequences (QSs) of mouse rG4s across vertebrate species**

The conservation statuses of the QSs underlying mouse rG4s were determined using whole-genome multiple alignments. For a given species, if the genomic region aligned with the mouse rG4 contains a putative quadruplex sequence (PQS), the species possesses a positionally conserved PQS; thus, the QS underlying the mouse rG4 is conserved. Vertebrate species are further grouped into representative evolutionary clades (in reverse-chronological order) to illustrate the correlations between evolutionary times and the QS/PQS conservation status. The member species in each clade and the estimated evolutionary divergence times are shown in Figure S2. rG4s are further grouped by their harboring gene regions (5′ UTR/CDS/3′ UTR) to highlight differences in their conservation status. The bar plot shows the overall distribution of the conservation statuses of all rG4s, regardless of their canonicality. The overlaid kernel distribution estimation plot shows the distribution of individual rG4s of different canonicalities.

**Figure S4 Divergence of the sequences of mouse rG4s across vertebrate species**

The evolutionary sequence divergence of mouse rG4s was determined using whole-genome multiple alignments. The sequence identities (using mouse rG4s as the references) of the genomic alignments from all species were calculated and averaged to produce the average sequence identity metric. rG4s without proper genomic alignments in all species were assigned an average sequence identity of 0.0; the corresponding bar plot is highlighted in green. Vertebrate species are further grouped into representative evolutionary clades (in reverse-chronological order) to illustrate the correlations between evolutionary times and sequence divergence. The member species of each group and the estimated evolutionary divergence times are shown in Figure S3. rG4s are further grouped by their harboring gene regions (5′ UTR/CDS/3′ UTR) to highlight differences in their conservation status. The bar plot shows the overall distribution of the sequence divergence of all rG4s, regardless of their canonicality. The overlaid kernel distribution estimation plot shows the distribution of individual rG4s of different canonicalities.

**Figure S5 The associations between sequence identities of CDS rG4s and rG4 canonicality**

Information from Figure 6 (Humans) and Figure S4 (Mice) is reorganized here to illustrate the association between sequence identities of CDS rG4s and rG4 canonicality. Violin plots show the distributions of average sequence identities of CDS rG4s of different canonicality. The “any” group includes all rG4s. Vertebrate species are further grouped into representative evolutionary clades (in reverse-chronological order) to illustrate the correlations between evolutionary times and sequence divergence. Statistical tests (Mann-Whitney) were performed to compare the sequence identity distributions between adjacent rG4 canonicality groups. The member species of each group and the estimated evolutionary divergence times are shown in Figure S1 (Human) and S2 (Mice). Overall, rG4s with canonicality values of 4 or 3 do not exhibit significant differences in sequence identity, whereas rG4s with canonicality values of 2, 1, and 0 tend to have progressively lower sequence identity. This pattern is more pronounced in earlier diverged species groups (Mammalia, Teterapoda, Vertebrata) compared to recently diverged groups (Primates/Glires, Euarchontoglires, Eutheria).

Statistical significance is denoted as: n.s. (*P* > 0.05), * (*P* ≤ 0.05), ** (*P* ≤ 0.01), *** (*P* ≤ 0.001), **** (*P* ≤ 0.0001).

**Figure S6 The associations between PQS conservation and the sequence identities of mouse rG4s**

Information from Figures S3 and S4 is combined here to illustrate the association between PQS conservation and the sequence divergence of mouse rG4s. Individual violin plots show the distributions of average sequence identities (from Figure S4), while different violin plots show the rG4s associated with different PQS conservation statuses (Figure S3). Vertebrate species are further grouped into representative evolutionary clades (in reverse-chronological order) to illustrate the correlations between evolutionary time and rG4 evolution. rG4s are further grouped by their harboring gene regions (5′ UTR/CDS/3′ UTR) to highlight differences in their conservation status. Under normal circumstances, higher PQS conservation might be correlated with lower sequence divergence. However, this type of correlation is not observed in some subplots (*e.g.*, x1, y1, y2, y3), suggesting that under specific circumstances (gene region and evolutionary times), sequence divergence and PQS conservation may not influence each other.

**Figure S7 Comparing the canonicality of mouse rG4s and the putative quadruplex sequences (PQS) on ancestral sequences**

The ancestral sequences of mouse rG4s in the Mammalian and Tetrapodal clades were reconstructed based on the multiple alignments of genomic sequences. A PQS search of the ancestral sequences was performed, and the canonicality of the PQSs was determined as described in Figure 5A. The plot does not include ancestral sequences that do not contain a PQS. **A.** Landscape of canonicality in reconstructed Mammalian and Tetrapodal ancestral PQSs, classified by the canonicality of their aligned mouse rG4s. **B.** and **C.** Comparison of canonicality between mouse rG4s and their aligned Mammalian (B) and Tetrapodal (C) ancestral PQSs.

**Figure S8 User interfaces of the rG4-seq Database application**

**A.** The rG4 List View interface provides a summary table of the rG4 detections and allows users to search for entries using a combination of criteria (*e.g.*, gene, transcripts, gene region, and structural motifs). **B.** The Genome Browser View interface displays the gene structures and locations of rG4 motifs using a genomic coordinate system. The compact list view below shows rG4s within the browsing window and is automatically updated. **C.** The rG4 Detail View pop-up window can be viewed by selecting the right-click menu on each rG4 entry. The window shows the details of each rG4, including basic information, the raw RTS signals in rG4-seq experiments, the sequence conservation status of the rG4 motif (only for human and mouse rG4s), and the results of secondary structure prediction under rG4-folding and rG4-unfolded conditions. Links to external genomic database resources such as the UCSC Genome Browser and Ensembl are also provided.

**Table S1 Statistics of genomes, transcriptomes, and density of rG4 detections in surveyed species**

**Table S2 Statistics of rG4s overlapping with predicted 3′ UTR miRNA binding sites**

**Table S3 Statistics of rG4s overlapping with splice junctions**
