# Supplementary material for: Differential Evolution of CDS and UTR Non-canonical RNA G-quadruplex Structures in Eukaryotic Transcriptomes
Source: Genomics Proteomics Bioinformatics. 2025 Sep 14;23(6):qzaf078. doi: 10.1093/gpbjnl/qzaf078 (PMC13198871; doi:10.1093/gpbjnl/qzaf078)
Supplement: qzaf078_Supplementary_Data [file qzaf078_supplementary_data.zip › table_S1.docx]

**Table S1 Statistics of genomes, transcriptomes, and density of rG4 detections in surveyed species**

|  | **Genome size** | **Genome  GC percent** | **Transcriptome  size** | **rG4s detected (G3+G2)** | | | **rG4s detected (G3+G2+Variant G2)** | | |
| --- | --- | --- | --- | --- | --- | --- | --- | --- | --- |
|  |  |  |  | **No. of detections** | **Per Mb genome** | **Per Mb transcriptome** | **No. of detections** | **Per Mb genome** | **Per Mb transcriptome** |
| Human | 3.1 Gb | 41.0 | 147.8 Mb | 20,332 | 6.6 | 137.6 | 28,375 | 9.2 | 192.0 |
| Mouse | 2.7 Gb | 41.5 | 128.4 Mb | 15,561 | 5.8 | 121.2 | 20,796 | 7.7 | 161.9 |
| Zebrafish | 1.4 Gb | 36.5 | 75.5 Mb | 1337 | 1.0 | 17.7 | 1568 | 1.1 | 20.8 |
| Fruit Fly | 143.7 Mb | 42.0 | 35.9 Mb | 4421 | 30.8 | 123.2 | 7131 | 49.6 | 198.7 |
| Nematode | 100.3 Mb | 35.5 | 32.0 Mb | 1537 | 15.3 | 48.0 | 2701 | 26.9 | 84.3 |
| Yeast | 12.1 Mb | 38.5 | 8.9 Mb | 890 | 73.6 | 99.8 | 1813 | 149.8 | 203.4 |
| *Plasmodium* | 23.3 Mb | 19.5 | 21.5 Mb | 737 | 31.6 | 34.3 | 2569 | 110.3 | 119.5 |
