# Supplementary material for: Differential Evolution of CDS and UTR Non-canonical RNA G-quadruplex Structures in Eukaryotic Transcriptomes
Source: Genomics Proteomics Bioinformatics. 2025 Sep 14;23(6):qzaf078. doi: 10.1093/gpbjnl/qzaf078 (PMC13198871; doi:10.1093/gpbjnl/qzaf078)
Supplement: qzaf078_Supplementary_Data [file qzaf078_supplementary_data.zip › table_S2.docx]

**Table S2 Statistics of rG4s overlapping with predicted 3′ UTR miRNA binding sites**

| **rG4 structural motifs** | **No. of rG4s overlapping targetScan predicted microRNA binding sites** | **Percentage** |
| --- | --- | --- |
| Human |  |  |
| G3 |  |  |
| Canonical/G3L1-7 | 149 | 8.86% |
| Long loop | 114 | 7.96% |
| G2 |  |  |
| Bulges | 244 | 3.96% |
| Two-quartet | 333 | 3.01% |
| Mouse |  |  |
| G3 |  |  |
| Canonical/G3L1-7 | 118 | 9.78% |
| Long loop | 59 | 7.41% |
| G2 |  |  |
| Bulges | 171 | 4.00% |
| Two-quartet | 322 | 3.47% |
| Fruit fly |  |  |
| G3 |  |  |
| Canonical/G3L1-7 | 1 | 3.33% |
| Long loop | 1 | 3.45% |
| G2 |  |  |
| Bulges | 28 | 0.80% |
| Two-quartet | 8 | 0.91% |
