# Supplementary figures and images for: Differential Evolution of CDS and UTR Non-canonical RNA G-quadruplex Structures in Eukaryotic Transcriptomes
Source: Genomics Proteomics Bioinformatics. 2025 Sep 14;23(6):qzaf078. doi: 10.1093/gpbjnl/qzaf078 (PMC13198871; doi:10.1093/gpbjnl/qzaf078)

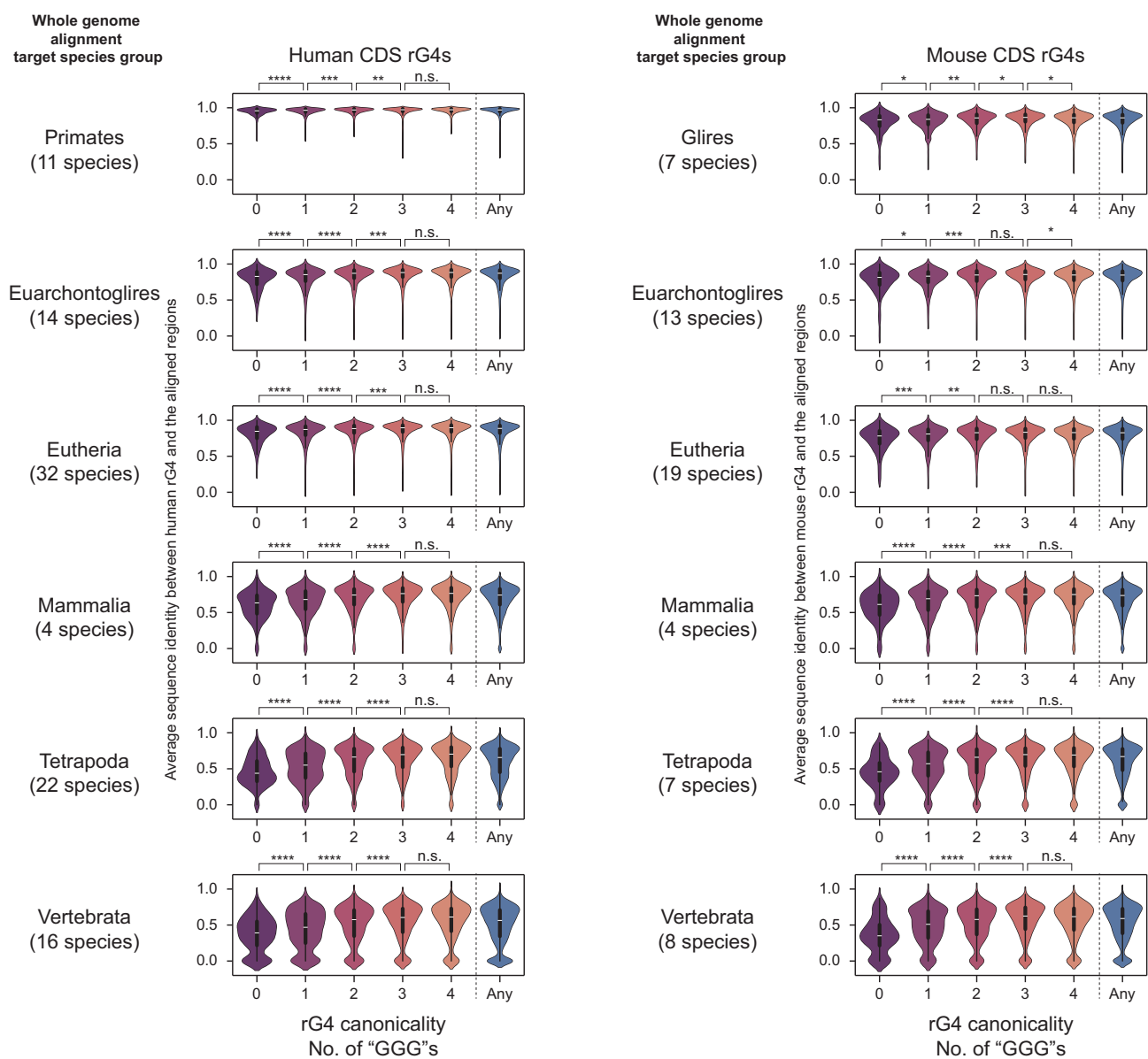

Supplement: qzaf078_Supplementary_Data [file qzaf078_supplementary_data.zip › Figure_S5.pdf]

Whole genome  
alignment  
target species group

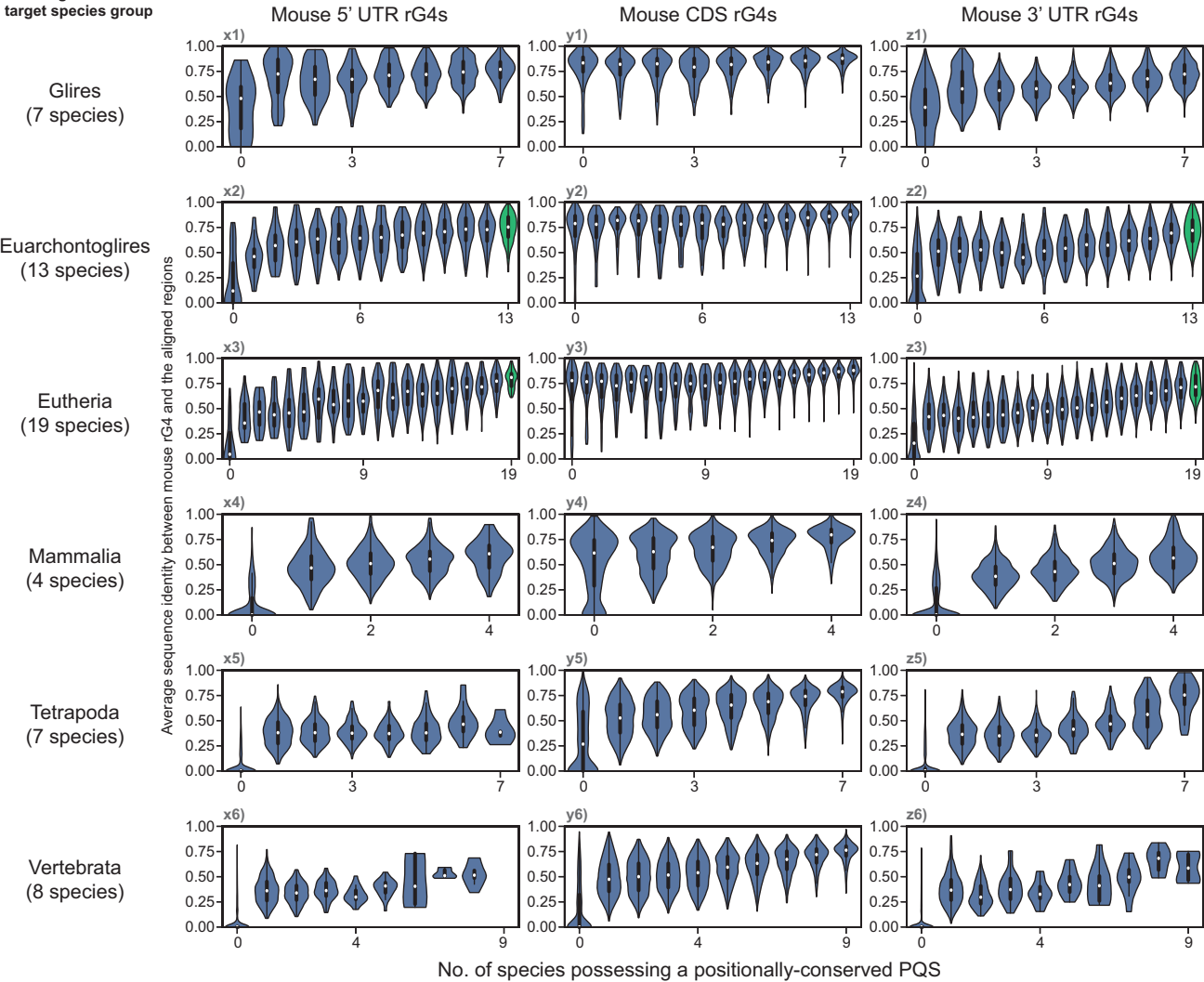

Supplement: qzaf078_Supplementary_Data [file qzaf078_supplementary_data.zip › Figure_S6.pdf]
